# Ecological Functions of Agricultural Soil Bacteria and Microeukaryotes in Chitin Degradation: A Case Study

**DOI:** 10.3389/fmicb.2019.01293

**Published:** 2019-06-20

**Authors:** Adam S. Wieczorek, Oliver Schmidt, Antonis Chatzinotas, Martin von Bergen, Antonie Gorissen, Steffen Kolb

**Affiliations:** ^1^Department of Ecological Microbiology, University of Bayreuth, Bayreuth, Germany; ^2^Department of Environmental Microbiology, Helmholtz Centre for Environmental Research-UFZ, Leipzig, Germany; ^3^German Centre for Integrative Biodiversity Research (iDiv), Leipzig, Germany; ^4^Department of Molecular Systems Biology, Helmholtz Centre for Environmental Research-UFZ, Leipzig, Germany; ^5^Faculty of Biosciences, Pharmacy and Psychology, Institute of Biochemistry, University of Leipzig, Leipzig, Germany; ^6^Department of Chemistry and Bioscience, University of Aalborg, Aalborg, Denmark; ^7^IsoLife BV, Wageningen, Netherlands; ^8^Microbial Biogeochemistry, RA Landscape Functioning, Leibniz Centre for Agricultural Landscape Research (ZALF), Müncheberg, Germany

**Keywords:** soil, microbiome, stable isotope probing, agriculture, soil carbon, food web, Ap horizon

## Abstract

Chitin provides a valuable carbon and nitrogen source for soil microorganisms and is a major component of particulate organic matter in agricultural soils. To date, there is no information on interaction and interdependence in chitin-degrading soil microbiomes. Since microbial chitin degradation occurs under both oxic and anoxic conditions and both conditions occur simultaneously in soil, the comparison of the active microbiome members under both conditions can reveal key players for the overall degradation in aerated soil. A time-resolved 16S rRNA stable isotope probing experiment was conducted with soil material from the top soil layer of a wheat-covered field. [^13^C_U_]-chitin was largely mineralized within 20 days under oxic conditions. *Cellvibrio*, *Massilia*, and several *Bacteroidetes* families were identified as initially active chitin degraders. Subsequently, *Planctomycetes* and *Verrucomicrobia* were labeled by assimilation of ^13^C carbon either from [^13^C_U_]-chitin or from ^13^C-enriched components of primary chitin degraders. Bacterial predators (e.g., *Bdellovibrio* and *Bacteriovorax*) were labeled, too, and non-labeled microeukaryotic predators (*Alveolata*) increased their relative abundance toward the end of the experiment (70 days), indicating that chitin degraders were subject to predation. Trophic interactions differed substantially under anoxic and oxic conditions. Various fermentation types occurred along with iron respiration. While *Acidobacteria* and *Chloroflexi* were the first taxa to be labeled, although at a low ^13^C level, *Firmicutes* and uncultured *Bacteroidetes* were predominantly labeled at a much higher ^13^C level during the later stages, suggesting that the latter two bacterial taxa were mainly responsible for the degradation of chitin and also provided substrates for iron reducers. Eventually, our study revealed that (1) hitherto unrecognized *Bacteria* were involved in a chitin-degrading microbial food web of an agricultural soil, (2) trophic interactions were substantially shaped by the oxygen availability, and (3) detectable predation was restricted to oxic conditions. The gained insights into trophic interactions foster our understanding of microbial chitin degradation, which is in turn crucial for an understanding of soil carbon dynamics.

## Introduction

Microbiomes in agricultural soils are fed by carbon and energy input from plant-derived organic residues, including root exudates. This input is partially transformed into the fungal and arthropod biomass (Gooday, 1990a,b; Martínez et al., 2009; Kramer et al., 2016). Together with plant residues, fungal and arthropod biomass contributes substantially to the pool of particulate organic matter which is turned over by the soil microbiome (Singh and Gupta, 1977; Scheu, 2002). Structural polymers form a major part of this pool of substrates, of which cellulose, hemicellulose, and chitin are more prone to microbial degradation than lignin. Unraveling agricultural and other soil microbial food webs that are associated with cellulose and complex plant litter degradation has been the goal of previous studies (e.g., El Zahar Haichar et al., 2007; Schellenberger et al., 2010; Kramer et al., 2016). However, the trophic interactions, dynamics, and ecological functions of members of a soil microbiome that degrades chitin await further elucidation. Chitin is the most abundant polysaccharide in terrestrial ecosystems after cellulose (Gooday, 1990a,b). It consists of β-1-4-linked N-acetylglucosamine (GlcNAc) residues and is a structural component of protists, arthropods, and fungi (Gooday, 1990a,b; Martínez et al., 2009). Fungal cell walls contain up to 25% chitin (Blumenthal and Roseman, 1957) and since fungi reach up to 60–90% of the microbial biomass in agricultural soils, they are thus the main source of chitin in such soils (Fernandez and Koide, 2012).

*Bacteria* have been recognized as the predominant chitin degraders and various species of *Actinobacteria, Proteobacteria,* and *Firmicutes* are considered to be important chitinolytic bacteria in soil (Gooday, 1990a,b; Beier and Bertilsson, 2013). However, our understanding of trophic interactions between chitinolytic and other soil microorganisms is solely based on information from pure or co-culture experiments as well as gene marker surveys (Gooday, 1990a,b; Jagmann et al., 2010; Beier and Bertilsson, 2013; Wieczorek et al., 2014), and an experiment that would directly reveal trophic interactions of the soil microbiome members involved in chitin degradation has yet to be completed.

Chitinolytic microorganisms use various and different extracellular enzymes to solubilize chitin, which may allow them to occupy different functional niches in regard to chitin breakdown. The enzymatic hydrolysis of chitin fibers by soil microorganisms is complex and requires the synergetic actions of different enzyme types. Exo- and endochitinases are the most important enzymes in chitin breakdown and hydrolyze chitin into oligomers of N-acetyl-glucosamine (Wild et al., 2018). Many bacterial chitinases are encoded by the gene *chiA,* which has been used in previous studies to detect chitinolytic microorganisms in the environment (Beier and Bertilsson, 2013; Cretoiu et al., 2013; Wieczorek et al., 2014). In addition, lytic polysaccharide monooxygenases (LPMO) break chitin chains by oxidative cleavage and increase the rate and efficiency of chitin degradation (Vaaje-Kolstad et al., 2010, 2013). N-acetyl-glucosamine dimers are taken up by soil microorganisms and are cleaved by β-N-acetylglucosaminidases to N-acetyl-glucosamine, which is further metabolized as a source of energy, carbon, and nitrogen (Gooday, 1990a; Keyhani and Roseman, 1999).

The aforementioned metabolic steps of microbial chitin breakdown also occur in agricultural soils of arable land which is considered to be largely oxic (Wieczorek et al., 2014). Nonetheless, anoxia occurs in microzones of such soils, as the oxygen distribution is heterogeneous and dynamic (Wagner et al., 1996; Or et al., 2007). Thus, contrasting energy-conserving microbial metabolisms occur at close proximity to each other and contribute to the degradation of biopolymers in agricultural soils (Schellenberger et al., 2010; Kramer et al., 2016). Hence, the availability of oxygen in a soil of arable agricultural land is a key environmental factor that determines the activity of different biopolymer-degrading microbial species.

In a previous study, we detected potential chitinolytic microorganisms with the gene marker *chiA* (encoding GH18 – glycoside hydrolase family – chitinases), which only allowed for a taxonomically limited identification of chitinolytic microorganisms and did not allow for the identification of trophically linked, non-chitinolytic microorganisms (Wieczorek et al., 2014). Therefore, the objective of the current study was to resolve the carbon flow path through the microbiome and to resolve the trophic interactions in an agricultural soil sample under oxic and anoxic conditions using an RNA-based stable isotope labeling approach with fungal chitin and soil material from a wheat-covered field in South Germany.

## Materials and Methods

### Sampling Site and Soil Properties

The sampling site is located on the research farm “Klostergut Scheyern” near Munich, Germany (48°30.0´N, 11°20.7´E). The upper 20-cm layer of aerated agricultural soil was sampled in April 2012 and processed within a week. The mean annual precipitation was 803 mm, with a mean annual temperature of 7.4°C (Wieczorek et al., 2014). The soil type was a Dystric Cambisol (FAO soil classification system) (Wieczorek et al., 2014). The C/N ratio was 6.9 ± 0.1. Soil pH (measured in water) was 6.6 ± 0.1, and the gravimetric water content was 21.9% (±1.0%). Ammonium, nitrate, and sulfate concentrations were below the detection limit of 0.1, or were 2.2 ± 0.2, and 1.2 ± 0 0.1 μmol g_soilDW_^−1^, respectively. Total amounts of iron and manganese were 103.4 ± 37.5 and 10.0 ± 6.7 μmol g_soilDW_^−1^, respectively.

### Soil Incubations and ^13^C-Labeling Experiments

Three hundred grams of freshly sampled soil were mixed with 750 ml of either sterile oxic or sterile anoxic water (ion-free distilled water) in rubber-stoppered 2-L flasks to prepare one oxic and one anoxic master slurry. The oxic and anoxic master slurries were flushed for 1 h with sterile air and sterile argon (100%, Rießner, Germany), respectively, and homogenized on an end-over-end shaker overnight at 5°C. Subsequently, the homogenized master slurries were divided into sets of six slurries each containing 110 ml in butyl rubber-stoppered 500-ml flasks. Slurries originating from the oxic master slurry were flushed with sterile air (oxic slurries) and those originating from the anoxic master slurry were flushed with sterile argon (anoxic slurries). Two of the oxic and anoxic slurries were supplemented with either 0.125 g of [^13^C_U_]-chitin ([^13^C]-chitin treatments), 0.125 g of [^12^C_U_]-chitin ([^12^C]-chitin treatments), or kept unsupplemented as control treatments (duplicate analysis). The [^13^C_U_]-chitin (>98 atom % ^13^C) and the [^12^C_U_]-chitin (1.1 atom % ^13^C) were purified from biomass of the fungus *Aspergillus niger* by IsoLife BV (Wageningen, Netherlands). The supplemented amount of chitin equals 5 mmol (44 mM) of carbon. Oxic and anoxic chitin treatments as well as the respective unsupplemented controls were incubated at 20°C on an end-over-end shaker for 70 days. The gas phase was exchanged in the oxic incubations every few days to keep the oxygen mixing ratios above 10%.

### Chemical Analyses

Liquid samples and gas samples were taken with sterile syringes. Liquid samples were centrifuged at 13,000 × *g* (Himac CT15E, Hitachi Koki Co., Ltd., Tokyo, Japan) for 15 min, and the supernatant was filtered (HPLC nylon filter, pore volume 0.2 μm, Infochroma, Zug, Switzerland). Organic acids and sugars were identified using high-performance liquid chromatography with ion exclusion chromatography (1,090 series II with UV detector, Hewlett Packard, Palo Alto, CA) (Wieczorek et al., 2014). Carbon dioxide, molecular hydrogen, and methane were measured with a gas chromatograph; the pH was measured with a pH electrode; and the ammonium, nitrate, and sulfate concentrations were assessed with ion chromatography, as described previously (Wieczorek et al., 2014).

For the analysis of ^12^CO_2_ and ^13^CO_2_ in the samples, gas chromatography-mass spectrometry (GC-MS) analysis was performed using a Perkin-Elmer GC Clarus 600 system with a Rtx®-1 capillary column (60 m × 320 μM). For GC-MS detection, an electron ionization system was operated with an ionization energy of 70 eV. Mass spectra were taken from 14 to 70 Da. Helium was used as carrier gas at a constant flow with 300 kPa, and an injection volume of 10 μl (split ratio 10:1) was employed manually by use of gastight syringes. Each sample was measured five times. The total amount of ^12^CO_2_ and ^13^CO_2_ was analyzed by extraction of the masses 44 and 45 followed by peak integration. The peak areas were corrected with ^12^CO_2_ and ^13^CO_2_ indoor air values and finally the ratio of the masses 45/44 was calculated. The soil moisture content was determined by weighing sieved soil before and after drying at 105°C for 48 h.

### RNA Stable Isotope Probing

To reduce sequencing effort and costs for the chitin, substrate duplicated microcosms were conducted and per time point and oxygen treatment only two SIP gradients were analyzed (one for the ^12^C control and one for ^13^C treatment). Nonetheless, RNA was extracted two times from duplicated slurries, i.e., two for ^12^C and two for ^13^C treatment, were equimolarly pooled and loaded on one gradient. Thus, for both oxygen treatments, from eight flasks, RNA was extracted at each time point. All as ^13^C-labeled identified OTUs were labeled at several time points. Although SIP gradients were not replicated at each time point, the dataset of labeled OTUs were replicated over time.

Nucleic acids were extracted from 0.4 g of soil slurry (Wieczorek et al., 2014), and RNA was obtained after digestion of DNA with DNase I (Fermentas, St. Leon-Roth, Germany). RNA SIP was performed according to published protocols (Whiteley et al., 2007; Schellenberger et al., 2010). Per analyzed time point of a treatment (Figures 2, 3), 200–500 ng of RNA was added to the gradient solution (buoyant density 1.79 g ml^−1^). Isopycnic centrifugation was performed to separate “heavy” potentially ^13^C-labeled RNA from “lighter” ^12^C-labeled RNA. RNA samples from oxic and anoxic chitin treatments were centrifuged in two separate runs using the same gradient solution to exclude gradient heterogeneity. Each gradient was separated into 10 fractions, and the buoyant density of each fraction was measured at 25°C (Supplementary Figure S1; Schellenberger et al., 2010). RNA was precipitated with ethanol, sodium acetate, and glycogen (Schellenberger et al., 2010) and resuspended in DNAse/RNAse free water (Gibco® Invitrogen, Germany). The RNA in each fraction was quantified with the Quant-iT RiboGreen RNA Assay Kit (Invitrogen, Germany) (Supplementary Figure S2). RNA from fractions with buoyant densities between 1.81 and 1.83 g ml^−1^ (“heavy” fractions) and RNA from fractions with buoyant densities between 1.76 and 1.77 g ml^−1^ (“light” fractions) were stored at −80°C and used for high-throughput sequencing of gene markers.

### High-Throughput Sequencing of Gene Markers

For testing if after the DNase digestion, DNA was completely removed, all RNA samples were subjected to PCR with primers and conditions used for amplifying cDNA. None of these PCRs revealed a product on a standard agarose gel. Thus, we concluded that those RNA samples were free of amplifiable DNA and proceeded with reverse transcription. Reverse transcription of RNA was performed with random hexamer primers (SuperScript III First-Strand Synthesis Supermix, Invitrogen, Karlsruhe, Germany) according to the manufacturer’s protocol. T4 GP32 protein (EURx Ltd., Gdansk, Poland) was added to a final concentration of 10 μg ml^−1^. Bacterial 16S rRNA gene transcripts were amplified from cDNA with primers 341F and 805R (Supplementary Tables S1, S2). A two-step amplification protocol was used to minimize the potential primer biases (Berry et al., 2011). In addition, 6-nucleotide (MID) instead of 10-nucleotide barcodes were used (Supplementary Figure S3, Supplementary Tables S3, S4). The first amplification round with untagged primers (25 cycles) was followed by a second amplification round using primers carrying adaptors, key, and MID (10 cycles). Amplicons were gel-purified, quantified, and pyrosequenced as previously described (Stacheter et al., 2013). Archaeal 16S rRNA gene, eukaryotic 18S rRNA gene, and *chiA* gene transcripts were amplified from cDNA using specific primers and amplification protocols (Supplementary Tables S1, S2), and the amplicons were sequenced by LGC Genomics GmbH with a MiSeq sequencer and V3 reagents (Illumina, Germany).

### Analysis of Pyrosequencing-Derived Data Analysis

Sequences were trimmed and quality filtered using ACACIA, such that erroneous homopolymers were corrected and low-quality reads were discarded from the dataset (Bragg et al., 2012). Prior to clustering, potential chimeras were filtered out (UCHIME algorithm implemented in USEARCH with the RDP Gold database release from 2016 for high-quality 16S rRNA gene reference sequences; Edgar et al., 2011). Information on the sequence numbers after each step is given in Supplementary Table S5. Sequences with a minimum length of 400 bp were analyzed using Jaguc2 (Nebel et al., 2011). In brief, Jaguc2 operates with average linkage clustering and pairwise alignments for calling operational taxonomic units (OTUs). Thus, this method is rather insensitive to sequencing errors such as insertions and deletions. Family-level OTUs were called with an average sequence similarity threshold of 87.5% (Yarza et al., 2010). Rarefaction analysis indicated that the sequencing effort was sufficient for the phylogenetic resolution as presented in the study (Supplementary Figure S4). OTUs were phylogenetically affiliated by local nucleotide BLAST using Jaguc2 against the latest SILVA SSU database release (SILVA 128; Yilmaz et al., 2014).

### Analysis of Illumina Sequencing-Derived Data

One million read pairs with a minimum length of 300 bp were provided by the commercial sequencing service in the *.fastq data format from all samples together. Paired-end forward and reverse reads were combined using BBMerge 34.48^1^ by the LGC Genomics GmbH and used for further analysis. Random subsamples (4,000 sequences each for archaeal 16S rRNA gene transcript and eukaryotic 18S rRNA gene transcript libraries; 2000 sequences each for *chiA* libraries) were taken using USEARCH as the computing time of Jaguc2 for the clustering increases exponentially (Nebel et al., 2011). A minimum of 1,221 reads per dataset remained for further analysis. Prior clustering barcodes as used in the pyrosequencing analysis were added to the fastq files *in silico*. After the addition of barcode sequences, sample files were merged into two fastq files, one file for the oxic and one for anoxic samples, respectively. Sequences were trimmed to 446 bp. Sequences with a minimum length of 400 bp were analyzed using Jaguc2. Two separate clustering runs were performed for oxic and anoxic treatments. Family-level OTUs were called with an average sequence similarity threshold of 87.5% for archaeal 16S rRNA gene transcript and eukaryotic 18S rRNA gene transcript sequences and 80% for *chiA* gene transcripts.

### Identification of ^13^C-Labeled Taxa by the Comparative Amplicon Pyrosequencing-Based Stable Isotope Probing Approach

Labeled bacterial 16S rRNA gene transcripts were identified following a modified comparative amplicon pyrosequencing-based stable isotope probing (CAP-SIP) approach modified compared to the one introduced by Dallinger and Horn (2014). Mean differences (±standard deviation) of the relative abundances per OTU in amplicon libraries derived from “heavy” fractions of [^13^C]- and [^12^C]-treatments at *t*_0_ were 0.04 (±0.19%) and 0.05% (±0.39%) for the oxic and anoxic treatments, respectively, indicating that artificial variations (caused by fractionation, amplicon generation, pyrosequencing, etc.) were minimal. These mean differences plus three times the respective standard deviations were calculated as a significance threshold (*T*: 0.61 and 1.22% for oxic and anoxic treatments, respectively). The threshold suggests a correct-detection rate of differences in 99.73% of the cases (Westgard et al., 1981). OTUs that met the criteria 1–3 and in most cases also criterion 4 were scored labeled at a certain time point *t_x_*: (1) *R*_a_ − *R*_b_ > *T* (*R*_a_ and *R*_b_ are the relative abundances of an OTU in the “heavy” fractions of the [^13^C]- and [^12^C]-treatment at *t_x_*, respectively); (2) *R*_a_ − *R*_c_ > *T* (*R*_c_ is the relative abundance of an OTU in the “light” fractions of the [^13^C]-treatment at *t_x_*); (3) *R*_a_ − *R*_d_ > *T* (*R*_d_ is the relative abundance of an OTU in the “heavy” fractions of the [^13^C]-treatment at *t*_0_); (4) *R*_cs_ > *T* [*R*_cs_ = (3 × *R*_a_ − *R*_b_ − *R*_c_ − *R*_d_)/3, i.e., the average relative CAP-SIP abundance (hereafter referred to as *R*_cs_ score)]. Raw relative abundances and *R*_cs_ scores of labeled OTUs are listed in Supplementary Tables S6, S7.

### Nucleotide Sequence Accession Numbers

All sequences obtained in the study (project number PRJEB14831) have been deposited under the sample accession numbers ERS1346811-ERS1346814 (18S rRNA reads of Eukaryota), ERS1262549-ERS1262552 (16S rRNA reads of *Archaea*), ERS1257899-ERS1258026 (16S rRNA reads of *Bacteria*), and ERS1346815-ERS1346846 (*chiA* reads) in the ENA archive of the European Bioinformatics Institute. Representative 16S rRNA sequences of labeled OTUs and representative transcript sequences of *chiA* OTUs were deposited in the ENA archive with accession numbers LT907870 to LT907902 and LT907849 to LT907862, respectively.

## Results and Discussion

### Chitin Degradation Under Oxic Conditions

**Figure 1:**
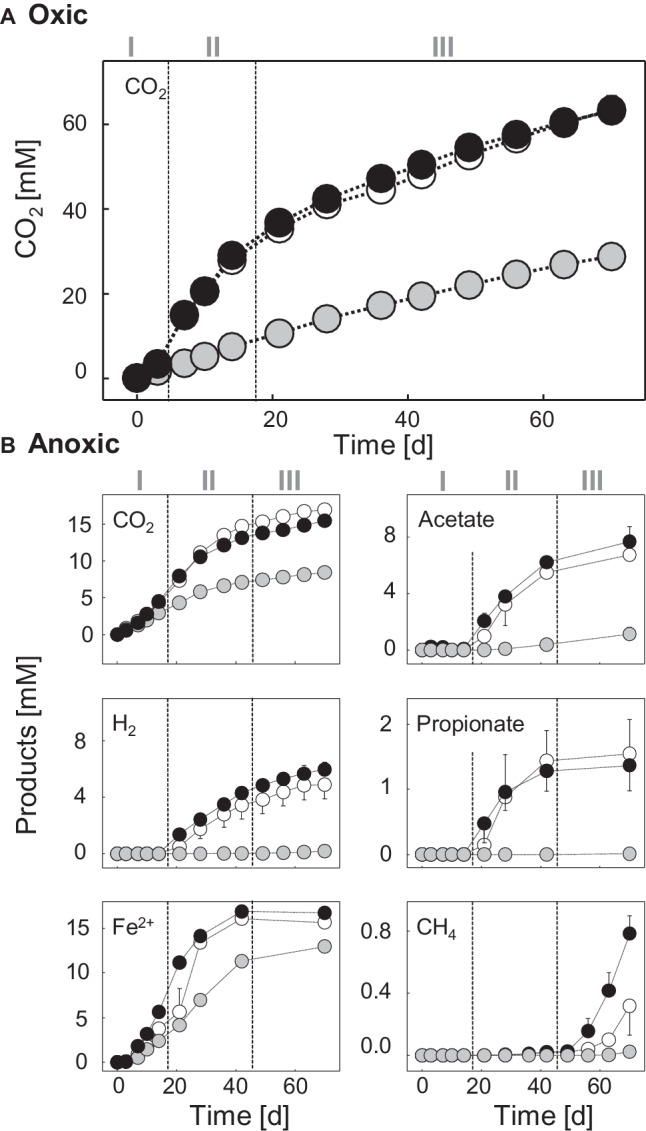
Products in soil slurries supplemented with [^13^C]-chitin, [^12^C]-chitin, and unsupplemented controls under oxic **(A)** and anoxic **(B)** conditions. ●, [^13^C]-chitin treatments; ○, [^12^C]-chitin treatments; ●, unsupplemented controls. Error bars, standard deviations of replicated microcosms (*n* = 2) are not shown when they were smaller than the symbol. Numerals indicate the different phases during chitin degradation.

CO_2_ production was stimulated after a short lag phase of about 3 days in chitin-supplemented treatments compared to unsupplemented controls (Figure 1A). This lag phase may reflect the exoenzymatic hydrolysis of the chitin prior to uptake and mineralization of the water-soluble breakdown products (Zhang and Lynd, 2004). Nevertheless, the stimulatory effect on the CO_2_ production indicated that chitinolytic microorganisms were prone to respond quickly to supplementation of fresh chitin. ^13^CO_2_ accumulation leveled off after 21 days (Supplementary Figure S5), suggesting that the supplemented [^13^C_U_]-chitin was largely consumed. At this time point, 54% of the chitin-derived ^13^C was recovered as ^13^CO_2_ (Table 1). A fraction of the residual ^13^C was likely assimilated during the synthesis of intra- and extracellular organic molecules (e.g., RNA or proteins) (Gooday, 1990a).

**Table 1:** Carbon and electron recoveries based on the assumption of the complete degradation of the supplemented [^13^C]-chitin.

	Oxic		Anoxic	
Day	Carbon recovery (%)	Electron recovery (%)	Carbon recovery (%)	Electron recovery (%)
3	3.0	n.d.	1.1	1.1
10	33.9	n.d.	0.7	1.2
21	54.0	n.d.	19.8	19.5
70	62.9	n.d.	57.9	54.1

n.d., could not be determined.

Ammonium can be released during chitin degradation (Wieczorek et al., 2014), and theoretically about 6 mM of ammonium could have been maximally released from the supplemented chitin. However, although most of the CO_2_ evolved originated from chitin, neither ammonium nor nitrate – as a product of ammonium oxidation catalyzed by nitrifiers – accumulated to considerably higher concentrations in chitin treatments compared to unsupplemented controls (Supplementary Figure S6A). This suggests that chitin-derived ammonium was readily assimilated for the synthesis of nitrogen-containing cell components, such as proteins, nucleic acids, and microbial cell wall components (Gooday, 1990a).

### Initial [^13^C]-Chitin Degraders Under Oxic Conditions

The relative abundances of *Gamma-* and *Betaproteobacteria* as well as *Bacteroidetes* were clearly higher in 16S rRNA amplicon libraries derived from cDNA of heavy fractions of the [^13^C]- compared to that of the [^12^C]-chitin treatments after 3 days of incubation (note that the relative abundances prior to the incubation were highly similar in heavy fractions of the [^13^C]- and [^12^C]-chitin treatments) (Supplementary Figure S7). These observations suggested that members of the aforementioned taxa were successfully labeled by the incorporation of [^13^C_U_]-chitin-derived carbon during synthesis of RNA within a few days.

**Figure 2:**
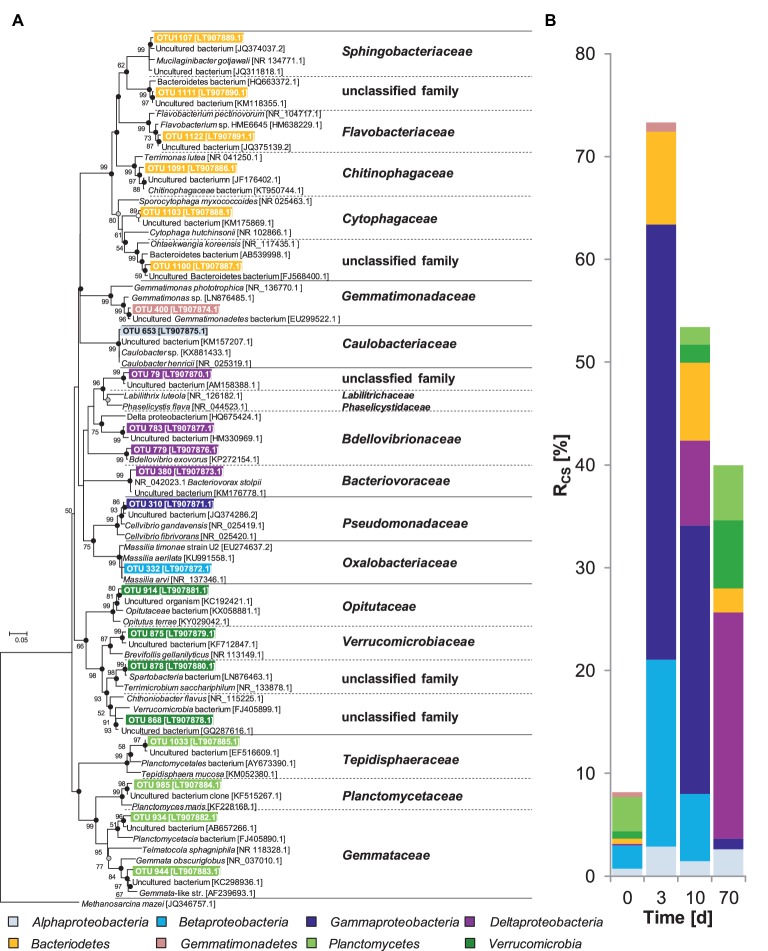
**(A)** Phylogenetic tree of 16S rRNA cDNA sequences from taxa ^13^C-labeled by assimilation of [^13^C]-chitin-derived carbon in soil slurries under oxic conditions and **(B)** corresponding *R*_cs_ scores. At *t*_0,_ relative abundance values are presented. The consensus tree was calculated with the maximum likelihood method and 1,000 bootstraps. Dots at nodes indicate confirmation of topology by neighbor joining (white circles) and maximum parsimony (grey circles) algorithms. Black circles indicate confirmation by both algorithms. Accession numbers are given in brackets. Scale bar, 5% evolutionary distance. *Methanosarcina mazei* (JQ346757.1) was used as the out-group. *R*_cs_ calculation is described in the “Materials and Methods” section.

OTU 310 was not detected prior to the incubation but showed the highest *R*_cs_ scores and, thus, was the most dominant of the labeled OTUs after 3 days (Figure 2, Supplementary Table S6). This suggests that OTU 310 was not active at the time point of sampling but was rapidly responding to the supplementation of chitin. Retrieved representative 16S rRNA gene transcript sequences of this OTU were closely related to *Cellvibrio* (98% identity to *Cellvibrio gandavensis* [NR_025419.1]), a genus of the *Pseudomonadaceae* (*Gammaproteobacteria*) that comprises several chitinolytic species (Humphry et al., 2003). It is likely that OTU 310 represented an important aerobic chitin degrader in the investigated soil, because it was predominantly ^13^C-labeled and was closely affiliated to a known chitinolytic genus.

The labeled OTU 332 had the second highest *R*_cs_ scores after 3 days of incubation (Figure 2, Supplementary Table S6). This OTU was affiliated with *Massilia* (*Betaproteobacteria*), a genus that comprises one cultured chitinolytic strain and its growth was stimulated in agricultural soils supplemented with chitin (Faramarzi et al., 2009; Cretoiu et al., 2014). Additionally, a *Massilia*-affiliated *chiA* genotype (OTU 4) was strongly ^13^C-labeled after 3 days (Supplementary Figure S8), which further underscores a contribution of *Massilia* species to the aerobic chitin degradation in soils.

Several less abundant OTUs were labeled after 3 days (Figure 2, Supplementary Table S6) and affiliated with the chitinolytic families *Sphingobacteriaceae, Flavobacteriaceae, Chitinophagaceae*, and *Cytophagaceae* of the *Bacteroidetes* phylum (Nakagawa, 2011; Yoon et al., 2012; McBride, 2014; Rosenberg, 2014). *Bacteroidetes* were also stimulated by supplemental chitin in studies with a different agricultural soil and in aquatic systems (Beier and Bertilsson, 2011; Cretoiu et al., 2014). Recently, it has been hypothesized that *Bacteroidetes* utilize alternative enzymatic mechanisms to solubilize biopolymers besides glycosidic hydrolases, the so-called “polysaccharide-utilizing loci” (PULs) (Naas et al., 2014; Berlemont and Martiny, 2015). PULs’ function in the synergistic solubilization and breakdown of chitin might be the degradation of solubilized chitin oligosaccharides (Martens et al., 2014). Our data eventually imply that members of known chitinolytic bacterial families *Gammaproteobacteria, Betaproteobacteria*, and *Bacteroidetes* conducted the initial chitin degradation under oxic conditions.

### Potential Secondary Chitin Degraders, Satellite Microbes, and Bacteriovorus Predators Under Oxic Conditions

*R*_cs_ scores for the *Gammaproteobacteria, Betaproteobacteria*, and *Bacteroidetes* decreased whereas those of the *Deltaproteobacteria, Planctomycetes*, and *Verrucomicrobia* increased after day 3 (Figure 2, Supplementary Table S6). The observed shift of labeled taxa is likely caused by different metabolic capabilities and functions of these taxa. In this regard, taxa that got labeled early most likely represent chitin degraders with high metabolic rates that thrive best under high substrate conditions (Lele and Watve, 2014; Lipson, 2015), and taxa that got labeled later during the incubation might represent either slow-growing chitin degraders potentially adapted to low substrate availability or were only indirectly involved in the mineralization of chitin-derived carbon (see discussion below).

In our experiment, *Planctomycetes* (*Gemmataceae, Planctomycetaceae*, and *Tepidisphaeraceae*) and *Verrucomicrobia* (*Opitutaceae, Verrucomicrobiaceae*, and unclassified *Verrucomicrobia* families) assimilated ^13^C. However, information about the physiology of *Planctomycetes* and *Verrucomicrobia* is limited to few cultured species. Some *Planctomycetes* utilize N-acetyl-glucosamine, the hydrolysis product of chitin (Rabus et al., 2002). Moreover, genomes of planctomycetes contain the *chiA* gene (Kulichevskaya et al., 2008; Guo et al., 2012; Ravin et al., 2018) and *Planctomycetes*-like *chiA* transcripts have been detected in our study (Supplementary Figure S8). *Planctomycetes* may also exhibit cellulolytic activity, and utilization of cellulose has been confirmed in a previous SIP study (Schellenberger et al., 2010; Kulichevskaya et al., 2012). Collectively, *Planctomycetes* are capable of biopolymer degradation, harbor enzymes necessary for chitin hydrolysis, utilize N-acetyl-glucosamine, and were labeled in our study, indicating that they might have been involved in chitin degradation in this experiment. However, the determination of chitinolytic capabilities among the *Planctomycetes* warrants further study. Similar to the *Planctomycetes, Verrucomicrobia* got ^13^C-labeled at days 10 and 70 in our experiments (Figure 2), suggesting that they were somehow involved in the mineralization of chitin-derived carbon. Only one study has suggested that members of the *Verrucomicrobia* might have chitinolytic activities (Chin et al., 1999), and further genome data suggest that they have metabolic potentials for polysaccharide degradation (Martinez-Garcia et al., 2012; Bai et al., 2016). Instead of being directly involved in chitin degradation, *Planctomycetes* and *Verrucomicrobia* may have been labeled due to the utilization of labeled carbon from exopolysaccharides (EPS) (Wang et al., 2015). Among taxa being clearly ^13^C-labeled in our study, the potential to produce EPS has been reported for the taxa *Caulobacteriaceae* (*Alphaproteobacteria*) and *Pseudomonadaceae* (*Gammaproteobacteria*) (Ravenscroft et al., 1991; Ueda and Saneoka, 2015). *Planctomycetes* and *Verrucomicrobia* could have also thrived on labeled bacterial cell envelope fragments that likely accumulated because of cell rupture of primary chitin degraders by predatory *Deltaproteobacteria* (Koval et al., 2013; see discussion below). A role as consecutive degraders of EPS and biofilm-associated polysaccharides is supported by the fact that members of both phyla have a broad repertoire of carbohydrate-active enzymes (Martinez-Garcia et al., 2012; Berlemont and Martiny, 2015; Bai et al., 2016).

The OTU 653 exhibited low but constant *R*_cs_ scores of 2–3% at all sampling days (Supplementary Table S6). It is closely related to *Caulobacter henricii* (*Caulobacteriaceae*) (Figure 2). No cultivated member of *Caulobacteriaceae* has been described as chitinolytic but several isolates can grow on chitin hydrolysis products in co-culture with chitinolytic partners (Eisenbeis et al., 2008; Abraham et al., 2014). Hence, we conclude that *Caulobacteriaceae* may have functioned as so-called “satellite microbes” that utilize hydrolysis products with saving metabolic investments for the synthesis of chitinolytic enzymes. By scavenging excess of chitin hydrolysis products, they might have accelerated the chitin degradation (Beier and Bertilsson, 2011; Bayer et al., 2013).

Labeled OTUs of the *Deltaproteobacteria* (OTUs 779, 783, 380, and 79) were affiliated with the *Bdellovibrionaceae, Bacteriovoracaceae,* and an uncultured family within the order *Myxococcales* (Figure 2, Supplementary Table S6). All of these taxa comprise well-known bacterial predators (Sockett, 2009; Berleman et al., 2014; Johnke et al., 2014). Thus, the detected *Deltaproteobacteria* in our experiment were more likely labeled by preying on labeled chitin degraders rather than by feeding on [^13^C_U_]-chitin and its hydrolysis products.

In addition to predation by bacteria, we had some evidence of predation by microeukaryotes. *Alveolata*, which were preying on cellulolytic bacteria in microcosms of the same soil (Chatzinotas et al., 2013), increased in relative abundances in 18S rRNA gene transcript amplicon libraries during the incubation (Supplementary Figure S9). However, a ^13^C-labeling of eukaryotic taxa could not be proven, since no 18S rRNA amplicons were obtained from the cDNA of heavy fractions from the [^13^C]-chitin treatments. Thus, eukaryotes might have played a role in the food web, but their ^13^C incorporation might have been not high enough for ^13^C-label detection in our experiment.

### Chitin Degradation Under Anoxic Conditions

The anaerobic degradation of chitin was separated into an initial phase (day 0 to day 14), an intermediary phase (days 14–42), and a final phase (days 42–70) based on the process data. During the initial phase, CO_2_ production was slightly stimulated in the chitin treatments compared with the unsupplemented controls (Figure 1B), and the production of [^13^C_U_]-chitin-derived ^13^CO_2_ was detectable on day 7 for the first time (Supplementary Figure S5). At the same time, the accumulation of ferrous iron started, whereas fermentation products were not detectable prior to day 21 (Figure 1B). Thus, chitin degradation obviously started soon after substrate supplementation under anoxic conditions and iron reducers likely contributed to early anaerobic chitin mineralization either by the degradation of carbohydrates derived from chitin-hydrolysis or by the scavenging of chitin fermentation products.

Molecular hydrogen (H_2_), acetate, and propionate accumulated in chitin treatments but not in unsupplemented controls after day 14 (Figure 1B). This suggests that chitin was degraded by fermenters and that the capacity for the production of these fermentation products exceeded a potential consumption by iron reducers during the secondary phase. Other fermentation products were detected in traces (butyrate, isobutyrate; Supplementary Figure S6B) or could not be detected (lactate, succinate, formate, and ethanol). These compounds were either not formed or effectively scavenged (e.g., by iron reducers).

In the final phase, the accumulation of fermentation products (including ^13^CO_2_) continued, albeit at a slower rate, until the end of the experiment, suggesting that residual chitin was present once the experiment stopped (Figure 1B, Supplementary Figure S5). The concentration of ferrous iron did not increase further in the final phase, most probably because of a depletion of the pool of ferric iron available. The formation of methane after 42 days is in accordance with the finding that in oxic soils, methanogens can be activated by long periods of anoxia (Küsel and Drake, 1994; Angel et al., 2012). However, judged by the low methane concentrations, methanogenesis was only a minor process in the anaerobic mineralization of chitin. Minor concentrations of ammonium accumulated in the anoxic chitin treatments, indicating that most of the chitin-derived ammonium was consumed, likely through assimilatory pathways (Supplementary Figure S6A). Hydrolytic products of chitin (i.e., N-acetylglucosamine and chitobiose) were not detected in either the oxic or the anoxic treatments, suggesting an efficient microbial consumption that resulted in low steady-state concentrations of these hydrolysis products (below the detection limit of 30 μM).

### Iron Reducers During Early Chitin Degradation Under Anoxic Conditions

**Figure 3:**
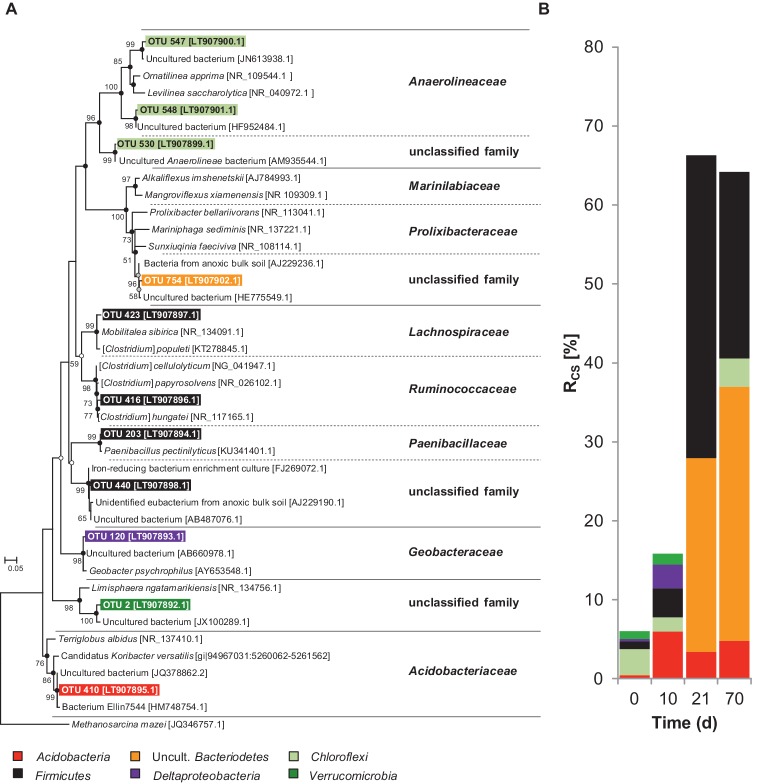
**(A)** Phylogenetic tree of 16S rRNA cDNA sequences from taxa labeled by assimilation of [^13^C]-chitin-derived carbon in soil slurries under anoxic conditions and **(B)** corresponding *R*_cs_ scores. At *t*_0,_ relative abundance values are presented. The consensus tree was calculated with the maximum likelihood method and 1,000 bootstraps. Dots at nodes indicate confirmation of topology by neighbor joining (white circles) and maximum parsimony (grey circles) algorithms. Black circles indicate confirmation by both algorithms. Accession numbers are given in brackets. Scale bar, 5% evolutionary distance. *Methanosarcina mazei* (JQ346757.1) was used as the out-group. *R*_cs_ calculation is described in the “Materials and Methods” section.

During the initial phase, ferrous iron but no fermentation products accumulated, pointing toward a contribution of iron reducers to chitin mineralization (Figure 1B). However, it cannot be concluded from the process data whether the iron reducers were (1) truly chitinolytic (i.e., excreted chitin-degrading exoenzymes), (2) satellite microbes that fed on sugars derived from chitin hydrolysis, or (3) scavengers of chitin-derived fermentation products. The latter is likely for OTU 120, which was labeled at day 10 and affiliated to the *Geobacteraceae*, a family that comprises iron reducers that use various fermentation products but not sugars as electron donor (Figure 3, Supplementary Table S7; Garrity et al., 2005). Such fermentation products may have been provided by chitinolytic fermenters potentially represented by the OTUs 410 and 203 that were labeled at day 10. OTU 410 was closely related to *Candidatus* “Koribacter versatilis,” a member of the *Acidobacteriaceae* that harbors genes encoding for chitin degradation (Ward et al., 2009). For other members of the *Acidobacteria*, biopolymer hydrolysis (incl. chitin) and fermentation of chitin hydrolysis products have been proven (Pankratov et al., 2012; Foesel et al., 2013; Kulichevskaya et al., 2014; Belova et al., 2018). OTU 203 was affiliated with *Paenibacillus*, a genus that comprises several species that can ferment chitin (Lee et al., 2004). Interestingly, some members of the *Acidobacteria* and *Paenibacillus* can use sugars as electron donors during iron reduction (Kulichevskaya et al., 2014; Li et al., 2014) and thus it might be possible that the labeled *Acidobacteria* and *Paenibacillus* phylotypes coupled chitin mineralization to iron reduction. However, both OTUs were also labeled at the end of the experiment when the pool of available ferric iron was depleted, indicating that these taxa were not restricted to iron reduction.

### Obligate Anaerobes Take Over After Prolonged Periods of Anoxia

After the initial phase, typical fermentation products accumulated (Figure 1) and the cumulative *R*_cs_ scores of all labeled taxa increased from 15.9% at day 10 to 66.4% at day 21 and remained constant afterward (64% after 70 days) (Figure 3, Supplementary Table S7). This suggests that chitin was readily fermented and chitin-derived ^13^C was increasingly assimilated. Interestingly, of the taxa that were labeled at day 10 (initial phase), only the *Paenibacillaceae* increased in abundance over time whereas the other initially labeled OTUs had lower *R*_cs_ scores or were not labeled anymore at day 21 or day 70 (Supplementary Table S7). In contrast, uncultured *Bacteroidetes* (OTU 754), *Lachnospiraceae* (OTU 423), and *Ruminococcaceae* (OTU 416) that were not labeled at day 10 made up for the dominant labeled taxa at days 21 and 70. All cultured isolates of *Lachnospiraceae* and *Ruminococcaceae* are obligate anaerobes (Rainey, 2009a,b), and the *Bacteroidetes*-affiliated OTU 754 was closely related (98% Blastn identity) to two obligate anaerobic isolates [strains PB90-2 (AJ229236) and XB45 (AJ229237)] from flooded paddy rice soils (Chin et al., 1999). Thus, our results point toward a shift in the anaerobic chitin-degrading microbial community after prolonged periods of anoxia most likely from facultative aerobes to obligate anaerobes.

The ^13^C incorporation by *Firmicutes*, i.e., *Ruminococcaceae* and *Lachnospiraceae,* agrees with pure culture-based observations that members of both families can degrade both chitin and cellulose (Evvyernie et al., 2000; Reguera and Leschine, 2001). Noteworthy, a *Firmicutes*-like *chiA* transcript (55% protein identity with *Clostridium beijerinckii*) was labeled (Supplementary Figure S10). In agreement with this finding, glycosyl hydrolase transcripts involved in chitin breakdown have been reported to be predominantly expressed by the *Firmicutes* in paddy soil slurries supplemented with rice straw (Wegner and Liesack, 2016). Taken together, the results emphasize the significant role of *Firmicutes* in the anaerobic degradation of chitin. Along with fermentation, ferric iron reduction was still ongoing until day 42. As *Geobacteriaceae* were not among the labeled phylotypes in the second half of the experiment, other iron-reducing taxa must have been active. The *Paenibacillus* OTUs were labeled during the whole experiment. *Paenibacillaceae* are known to be chitinolytic and capable of ferric iron reduction (Lee et al., 2004; Raza and Shen, 2010; Li et al., 2014). Thus, we think that *Paenibacillaceae* were partly responsible for the anaerobic mineralization of chitin and the observed ferric iron reduction in the second half of the experiment. At the end of the experiment, uncultured *Bacteroidetes* was the most abundant labeled phylum followed by *Firmicutes, Acidobacteria*, and *Chloroflexi* suggesting these were the primary chitin degraders in the second half of the experiment (Figure 3, Supplementary Table S7). Consistently, this phase of anaerobic chitin degradation was characterized by a substantial decrease of the ferric iron reduction rate.

### Functions of Archaea in Chitin Degradation

In agreement with the observed methane formation toward the end of the anoxic incubation (Figure 1), methanogenic *Archaea* (*Methanomicrobia* and *Methanobacteria*) were detected (Supplementary Figure S11). However, their relative abundances in libraries prepared from pooled PCR products derived from light fractions of [^13^C]- and [^12^C]-chitin treatments were low (Supplementary Figure S11) and no PCR products were obtained from the cDNA of heavy fractions with *Archaea*-specific primers. Nonetheless, in the amplicon libraries obtained with *Bacteria*-specific primers (Supplementary Figure S7), archaeal 16S rRNA gene sequences [98% Blastn identity with *Methanobacterium flexile* (NR_116276.1)] were present in the heavy fractions of ^13^C-treatments whereas no sequences were detected in the heavy fractions of ^12^C-chitin treatments. Thus, we conclude that methanogenic *Archaea* have assimilated some [^13^C_U_]-chitin-derived carbon by consumption of labeled metabolic products (i.e., CO_2_ and/or acetate) produced by obligate anaerobic chitin degraders and satellite microbes. However, note that the labeling was primed by a rather artificially prolonged anoxia and thus, conclusions regarding ecological functions of *Archaea* in chitin degradation based in soil on our data remain questionable.

### Weakly ^13^C-Labeled Bacterial Taxa

*Gemmatimonadetes* were labeled under oxic conditions whereas *Chloroflexi* were labeled under anoxic conditions albeit with low *R*_cs_ scores (Figures 2, 3, Supplementary Tables S6, S7). Our experimental data and literature knowledge are not conclusive enough to resolve their functions and trophic role in chitin degradation. Nonetheless, we conclude that based on their consistent low R_sc_ scores, *Gemmatimonadetes* and *Chloroflexi* were of minor importance.

### General Trends of Collective *R*_cs_ Scores

The sum of *R*_cs_ scores of all [^13^C]-labeled taxa under oxic conditions was highest after 3 days and decreased over time (Figure 2). This finding is in accordance with the observed rapid degradation of chitin (Figure 1A, Table 1). Under anoxic conditions, the trend in *R*_cs_ scores was opposite (Figure 3) but fitting well to the observed slower degradation of chitin and the increasing metabolic activity over time (Figure 1B).

### Final Conclusions

**Figure 4:**
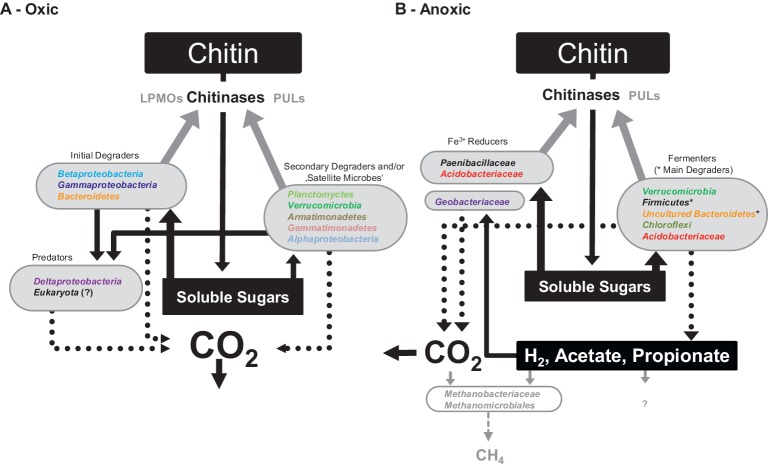
‘Conceptual model of soil microbiome interactions when degrading chitin under oxic (A) and anoxic (B) conditions based on experimental observations made in our study. Dotted lines represent carbon fluxes and/or synthesis of enzymes. Solid black lines and arrows represent substrate and metabolite transfer to the respective organisms. Grey shaded information represent likely but not experimentally well-proven information. Colors of taxa follow the same color code as used in Figures 2, 3.

The data collected in this study were combined to reconstruct a hypothetical model of the processes and linked microbial taxa during the aerobic and anaerobic degradation of chitin in the investigated soil (Figure 4). To minimize sequencing effort and costs for the chitin substrate, our experiment was duplicated and SIP gradients were done from pooled RNA from both duplicates per time point and oxygen treatment. Previous studies revealed low technical variability (Morawe et al., 2017; Chaignaud et al., 2018). All labeled OTUs occurred at two or more time points. Hence, the conclusion that they were ^13^C-labeled is based on at least two replicated observations over time. Chitin was likely enzymatically solubilized by endo- and exo-chitinases, and potentially also by the activity of LPMOs and PULs. Few key chitinolytic taxa, i.e., *Pseudomonas* and *Massilia*, were responsible for most of the chitin degradation under oxic conditions. Furthermore, several family taxa of *Bacteroidetes* with known chitinolytic representatives incorporated substantial amounts of [^13^C_U_]-chitin-derived carbon, underlining the ecological importance of *Bacteroidetes* for degradation of complex polysaccharides in soils. The role of PUL-harboring *Bacteroidetes* in the synergistic breakdown of chitin might be the degradation of solubilized chitin oligosaccharides as has previously suggested (Martens et al., 2014). Chitin hydrolysis products were not detected indicating that polymer hydrolysis was rate limiting. In addition to primary chitin degraders, “satellite microbes” were potentially active in keeping the concentrations of chitin hydrolysis products at low levels. The growth of primary consumers and “satellite microbes” allowed for predation by bacteriovorus *Bacteriovoraceae, Bdellovibrionaceae*, and uncultured *Myxococcales* that incorporated ^13^C from their prey cells. How *Planctomycetes* and *Verrucomicrobia* incorporated ^13^C is difficult to answer based on our data. However, we think that their main ecological function was degradation of complex heteropolysaccharides in EPS and cell walls (Kulichevskaya et al., 2007; Wang et al., 2015). Nonetheless, there is increasing evidence that members of aerobic *Planctomycetes* are chitinolytic microorganisms in wetland soils (Ivanova et al., 2018; Dedysh and Ivanova, 2019). Hence, they might have been less relevant but active chitin degraders also in our experiment.

Under anoxic conditions, chitin was readily degraded but its degradation was slower compared to that under oxic conditions, which might be partially explained by inactivity of LPMOs as they require oxygen for their enzymatic mechanism (Vaaje-Kolstad et al., 2010, 2013). Overall, different members of the soil microbiome were labeled by the assimilation of [^13^C_U_]-chitin-derived carbon under anoxic conditions. The initial anaerobic degradation of chitin was characterized by a low accumulation of fermentation products and a low labeling ratio (i.e., a low collective *R*_cs_ score). While *Acidobacteria* and *Chloroflexi* were initially labeled, *Firmicutes* and uncultured *Bacteroidetes* were predominantly labeled, suggesting that the latter two bacterial phyla were mainly responsible for chitin degradation and provided substrates for iron reducers. The fermentation products H_2_/CO_2_ and acetate were likely consumed by hydrogenotrophic methanogens that assimilated additionally acetate for anabolic reactions. No evidence for prey-predator or saprotrophic interactions was observed in the course of the experiment under anoxic conditions. However, literature indicates that anaerobic predatory microeukaryotes occur in soil (Murase and Frenzel, 2007; Murase et al., 2014). Thus, our experiment might have failed to detect them because our approach was insensitive.

Our data indicated (1) that hitherto unrecognized *Bacteria* were involved in the chitin-degrading food web of an agricultural soil, (2) that trophic interactions of the chitin-degrading microbial food web were substantially shaped by the oxygen availability, and (3) that predation was restricted to oxic conditions. The functional redundancy of the soil microbiome and the catabolic diversity likely enable continued biopolymer degradation independent of oxygen availability. A rapid decomposition of chitin to CO_2_ as found in the oxic treatments with pure chitin is in line with previous studies addressing chitin degradation in aerated top soil (Fernandez and Koide, 2012), suggesting that chitin is not as recalcitrant as it is sometimes believed to be (Godbold et al., 2006; Langley et al., 2006). The insights we gained into the trophic interactions of a chitin-degrading microbiome improve the understanding of turnover dynamics of chitin in soil. Soil chitin degradation is driven by complex microbiome of which members fulfill certain functional steps and together facilitate chitin mineralization.

## Author Contributions

SK designed the study and wrote together with AW and OS the manuscript. AW conducted the experimental work. AC and MB supported the interpretation of data and contributed to the final manuscript version. AG developed a laboratory procedure to produce high-quality [^13^C_U_]-chitin from a fungal strain.

### Conflict of Interest Statement

AG was employed by and director of the company IsoLife BV (Netherlands).The remaining authors declare that the research was conducted in the absence of any commercial or financial relationships that could be construed as a potential conflict of interest.

## Footnotes

^1^http://bbmap.sourceforge.net/
