# Supplementary material for: Ecological Functions of Agricultural Soil Bacteria and Microeukaryotes in Chitin Degradation: A Case Study
Source: Front Microbiol. 2019 Jun 20;10:1293. doi: 10.3389/fmicb.2019.01293 (PMC6596343; doi:10.3389/fmicb.2019.01293)
Supplement: Supplementary file 1 [file Data_Sheet_1.PDF]

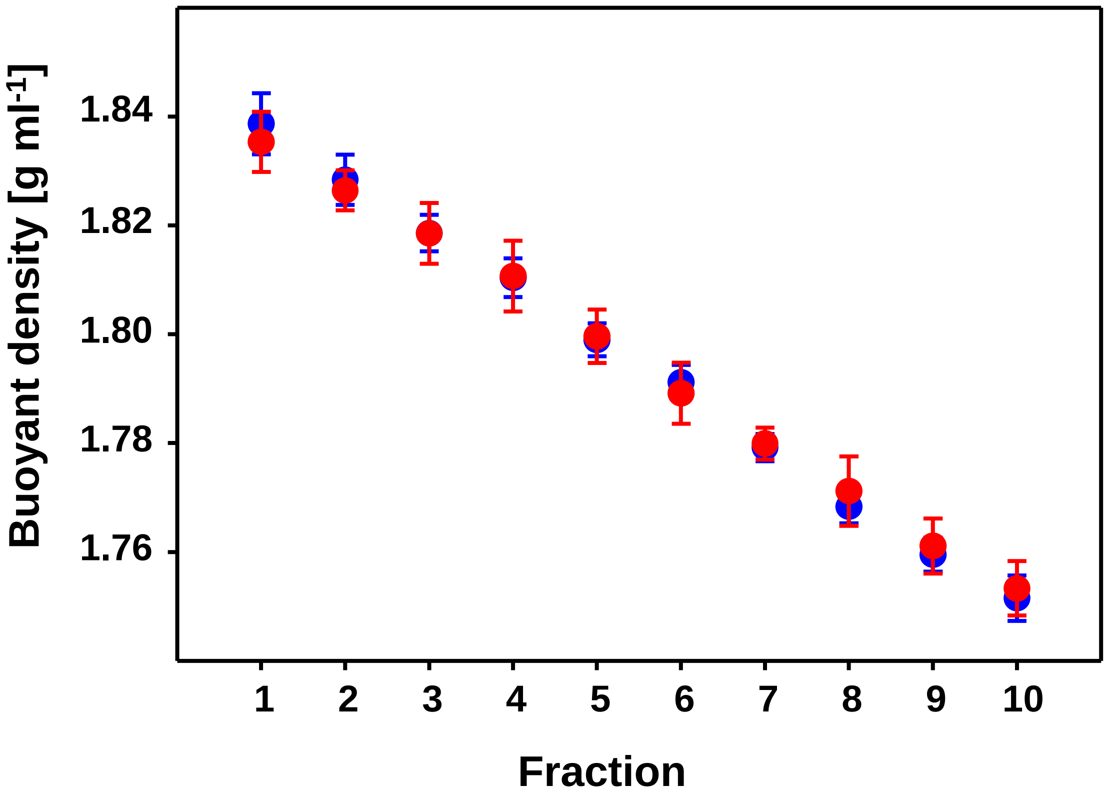

Figure S1. Buoyant density of gradient solution in fractions at 25°C. Blue circles, oxic samples. Red circles, anoxic samples. The density of each fraction was measured at 25°C (Manefield *et al*., 2002).

Reference:

Manefield, M., Whiteley, A.S., Griffith, R.I., and Bailey, M. (2002) RNA stable isotope probing, a novel means of linking microbial community function to phylogeny. *Appl Environ Microbiol* 68, 5367–5373.

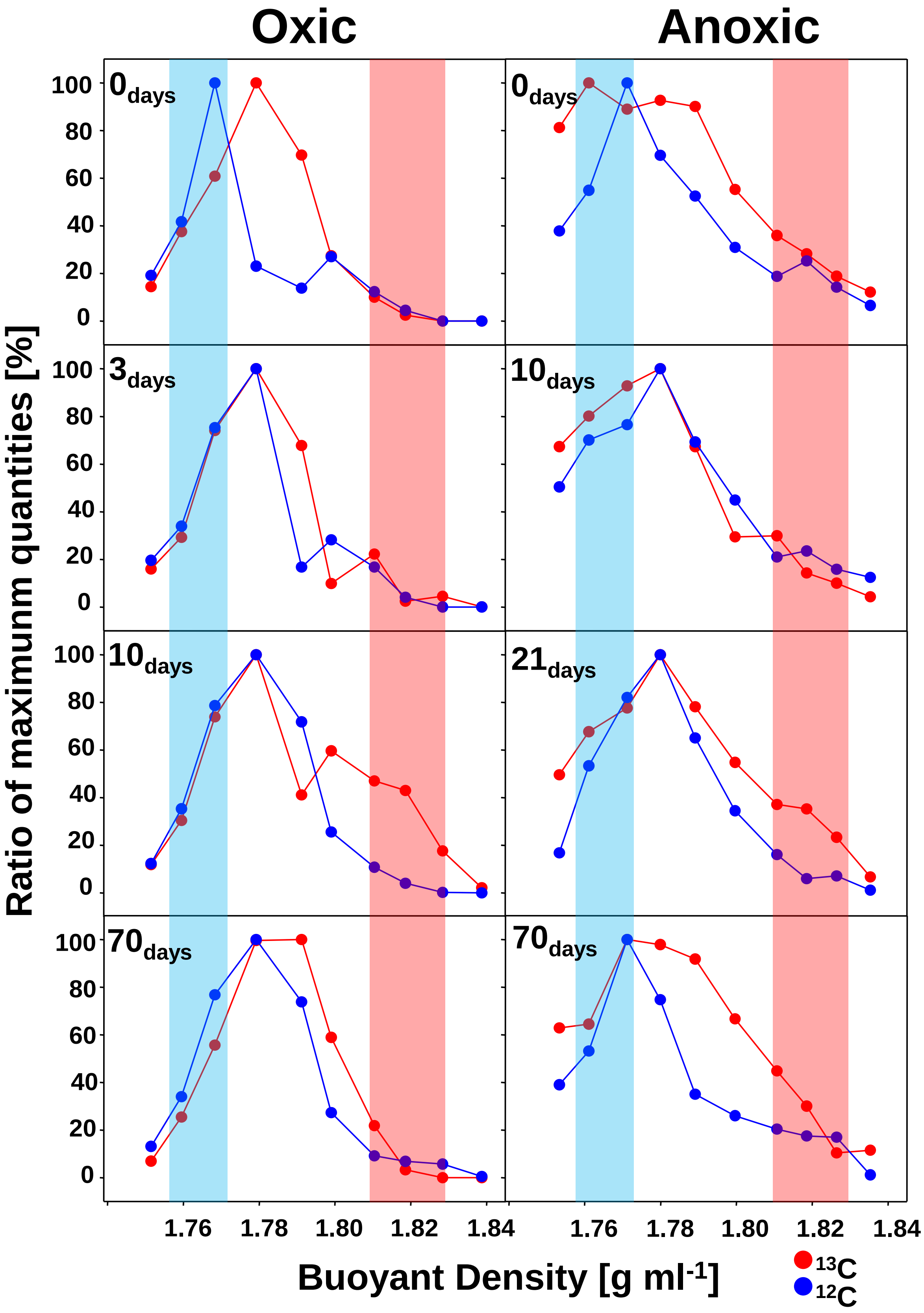

Figure S2. RNA distribution in the fractions 1-10. Blue transparent boxes ,´light´ fractions; red transparent boxes, ´heavy´ fractions representing unlabeled and labeled RNA, respectively.

Table S1. List of primers used in this study. Primer sets for the phylogenetic analysis of 16S rRNA transcripts of *Bacteria* and *Archaea* by next generation technologies were chosen as suggested by Klindworth *et al.*, 2013[a].

Target Gene	Direction	Primer Name ´Trivial´	Primer Name Specific[b]	Sequence 5´-3´	Reference
**16S rRNA**	Forward	Bakt_341F	S-D-Bact-0341-b-S-17	CCT ACG GGN GGC WGC AG	Muyzer *et al*., 1998
Bacteria	Reverse	Bakt_805R	S-D-Bact-0785-a-A-21	GAC TAC HVG GGT ATC TAA TCC	Herlemann *et al*., 2011
**16S rRNA**	Forward	A519F	S-D-Arch-0519-a-S-15	CAG CMG CCG CGG TAA	Wang and Qian 2009
Archaea	Reverse	Arch1017R	S-D-Arch-1041-a-A-18	GGC CAT GCA CCW CCT CTC	Yoshida *et al*., 2005
**18S rRNA**	Forward	TAReuk454FWD1	-	CCA GCA SCY GCG GTA ATT CC	Stoeck *et al*., 2010
Eukaryota	Reverse	TAReukREV3	-	ACT TTC GTT CTT GAT YRA	Stoeck *et al*., 2010
chiA	Forward	ChiA_F2	-	CGT GGA CAT CGA CTG GGA RTW YCC	Hobel *et al*., 2005
	Reverse	ChiA_R2	-	CCC AGG CGC CGT AGA RRT CRT ARS WCA	Hobel *et al*., 2005

[a]Klindworth, A., Pruesse, E., Schweer, T., Peplies, J., Quast, C., Horn, M., et al. (2013). Evaluation of general 16S ribosomal RNA gene PCR primers for classical and next-generation sequencing-based diversity studies. *Nucleic Acids Res.* 41, e1. doi: 10.1093/nar/gks808
[b]Alm, E.W., Oerther, D.B., Larsen, N., Stahl, D.A., and Raskin, L. (1996). The Oligonucleotide Probe Database. *Appl. Environ. Microbiol.* 62, 3557-3559.

References:

Herlemann, D.P.R., Labrenz, M., Jürgens, K., Bertilsson, S., Waniek, J.J., and Andersson, A.F. (2011). Transitions in bacterial communities along the 2000 km salinity gradient of the Baltic Sea. *ISME J.* 5, 1571–1579. doi: 10.1038/ismej.2011.41

Hobel, C.F.V., Marteinsson, V.T., Hreggvidsson, G.O., and Kristjánsson, J.K.(2005). Investigation of the microbial ecology of intertidal hot springs by using diversity analysis of 16S rRNA and chitinase genes. Appl. Environ. Microbiol. 71, 2771–2776. doi: 10.1128/AEM.71.5.2771-2776.2005

Yoshida, N., Yagi, K., Sato, D., Watanabe, N., Kuroishi, T., Nishimoto, K., et al. (2005). Bacterial communities in petroleum oil in stockpiles. J. Biosci. Bioeng. 99, 143–149. doi: 10.1263/jbb.99.143

Muyzer, G., Brinkhoff, T., Nübel, U., Santegoeds, C.M., Schäfer, H., and Wawer, C. (1998). “Denaturing gradient gel electrophoresis (DGGE) in microbial ecology.” in Molecular Microbial Ecology Manual, 3rd edn., eds A.D.L. Akkermans, J.D. van Elsas and F.J. de Bruijn (Dordrecht, The Netherlands: Kluwer Academic Publishers), 1–27.

Stoeck, T., Bass, D., Nebel, M., Christen, R., Jones, M. D., Breiner, H.-W., et al. (2010). Multiple marker parallel tag environmental DNA sequencing reveals a highly complex eukaryotic community in marine anoxic water. Mol. Ecol. 19, 21–31. doi: 10.1111/j.1365-294X.2009.04480.x

Wang, Y., and Qian, P.Y. (2009). Conservative fragments in bacterial 16S rRNA genes and primer design for 16S ribosomal DNA amplicons in metagenomic studies. PLOS ONE 4, e7401. doi: 10.1371/journal.pone.0007401

Table S2. Thermoprotocols for the amplification of 16S rRNA transcripts of *Bacteria* and *Archaea*, 18S rRNA transcripts of *Eukaryota* and transcripts of the structural gene marker *chiA*. Table S1, details on primers.

	Bacteria Bakt_341F/ Bakt_805R			***Archaea*** A519F/ Arch1017R			***Eukaryota*** TAReuk454FWD1/ TAReukREV3			***chiA*** ChiA_F2/ ChiA_R2		
	°C	s		°C	s		°C	s		°C	s	
Initial Denaturation	95	300		95	300		95	300		95	180	
Denaturation	95	40	25x	95	60	30x	95	60	30x	95	45	30x
Annealing	55	120		58	60		48	60		42	45	
Elongation	72	60		72	60		72	60		72	90	
Final-Elongation	72	420		72	300		72	300		72	300	

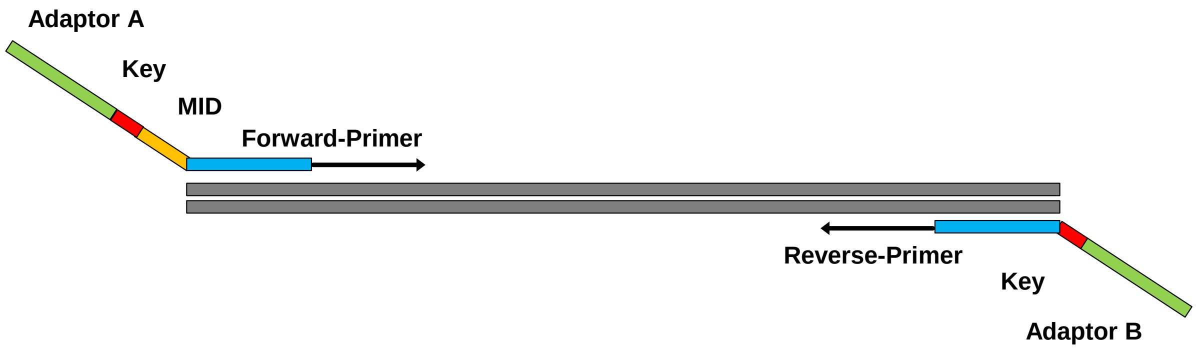

Figure S3. Primer setup for pyrosequencing of *Bacteria* 16S rRNA gene cDNA.

Table S3. Primer for pyrosequencing of *Bacteria* 16S rRNA gene cDNA.

	Pyrosequencing Primer Design			
	Adaptor	Key	MID[a]	Template specific[b]
Forward	5´-CCATCTCATCCCTGCGTGTCTCCGAC	TCAG	NNNNNN	(Bakt_341F)-3´
Reverse	5´-CCTATCCCCTGTGTGCCTTGGCAGTC	TCAG	-	(Bakt_805R)-3´

[a]Table S4 for MID sequences.
[b]Table S1 for primer sequences.

Table S4. MID sequences in primers for pyrosequencing of *Bacteria* 16S rRNA

		**Oxic**			**Anoxic**	
		**Heavy**	**Light**		**Heavy**	**Light**
**[^{13}C]-chitin**	t_0	5´-**ACGAGC**-3´	5´-**ACTATC**-3´	t_0	5´-**TAGCAC**-3´	5´-**TAGTGC**-3´
	t_1	5´-**ACTCGC**-3´	5´-**AGCGTC**-3´	t_3	5´-**TATCGC**-3´	5´-**TCACGC**-3´
	t_3	5´-**AGCTAC**-3´	5´-**AGTCAC**-3´	t_5	5´-**TCTAGC**-3´	5´-**TCTCAC**-3´
	t_{12}	5´-**ATACTC**-3´	5´-**ATATAC**-3´	t_{12}	5´-**TGACAC**-3´	5´-**TGCATC**-3´
**[^{12}C]-chitin**	t_0	5´-**ATCATC**-3´	5´-**ATCTGC**-3´	t_0	5´-**TGTCTC**-3´	5´-**TGTGAC**-3´
	t_1	5´-**ACACTG**-3´	5´-**AGCACG**-3´	t_3	5´-**TACACG**-3´	5´-**TACGTG**-3´
	t_3	5´-**AGTATG**-3´	5´-**ATAGTG**-3´	t_5	5´-**TACTAG**-3´	5´-**TAGCTG**-3´
	t_{12}	5´-**TACAGC**-3´	5´-**TAGATC**-3´	t_{12}	5´-**TATATG**-3´	5´-**TATCAG**-3´

Table S5. Sequence numbers after each step of sequence preparation prior clustering into family level OTUs. Raw reads of the oxic and anoxic libraries were converted into the fastq format and pooled into one fastq file, respectively.

	**Number of Sequences**	
	**Oxic**	**Anoxic**
Pooled Raw FastQ Files	126740	106712
Key Removed	126740	106712
Trimmed to 446bp	126721	106709
Quality Checked[a]	122305	103029
Chimeras Removed[b]	99456	87060

[a] with ´ACACIA´, i.e. erroneous homopolymers were corrected and low quality reads were discarded from the dataset (Bragg *et al.*, 2012).
[b] potential chimeras were filtered out (UCHIME´ algorithm implemented in ´USEARCH´ with the latest RDP Gold database for high quality 16S rRNA gene reference sequences (Edgar et al., 2011). See material and methods for further details.

References

Bragg, L., Stone, G., Imelfort, M., Hugenholtz, P., and Tyson, G. W. (2012). Fast, accurate error-correction of amplicon pyrosequences using Acacia. Nat. Methods 9, 425–426. doi: 10.1038/nmeth.1990

Edgar, R. C., Haas, B. J., Clemente, J. C., Quince, C., and Knight, R. (2011). UCHIME improves sensitivity and speed of chimera detection. *Bioinformatics* 27, 2194–2200. doi: 10.1093/bioinformatics/btr381

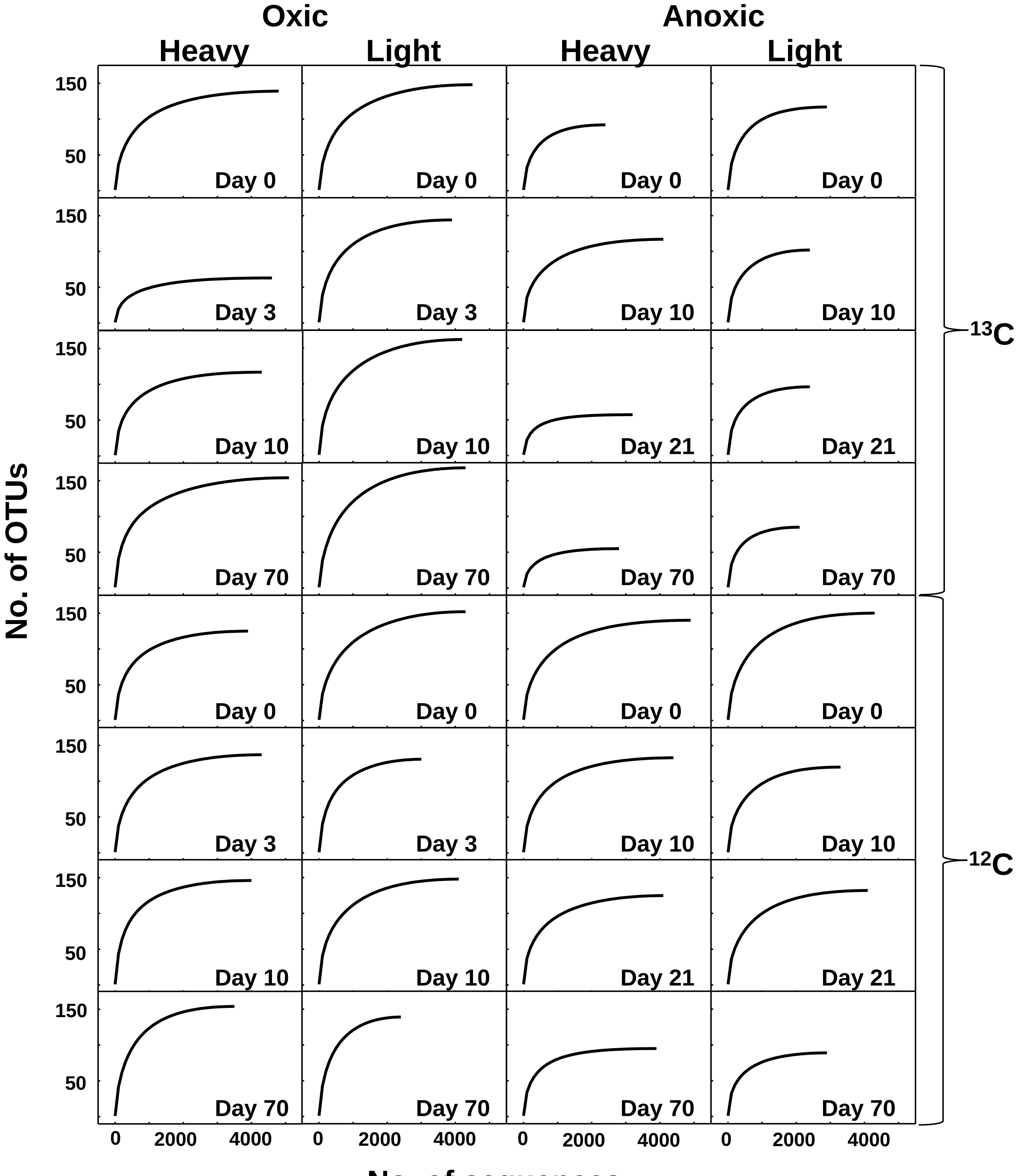

Figure S4. Rarefaction analysis of bacterial 16S rRNA sequences. OTUs were called based on a similarity cut-off of 87.5%. The program ‘aRarefact‘ was used to calculate the curves according to the Hulbert rarefaction (Hulbert 1971). Singletons were omitted in the analysis.

Reference

Hulbert, S.H. (1971). Nonconcept of species diversity-critique and alternative parameters. *Ecology* 52, 577-586. doi: 10.2307/1934145

Table S6. Relative abundances and calculated R_{CS} scores of labeled OTUs under oxic conditions.[a]

Phylum/Class		Family	OTU	[^{13}C]-chitin Day 0 H	Day 0 L	Day 3 H	Day 3 L	Day 10 H	Day 10 L	Day 70 H	Day 70 L	[^{12}C]-chitin Day 0 H	Day 0 L	Day 3 H	Day 3 L	Day 10 H	Day 10 L	Day 70 H	Day 70 L	R_{CS} score Day 3	R_{CS} score Day 10	R_{CS} score Day 70
Bacteriodetes		***Cytophagaceae***	**1100**	0.0	0.2	1.2	0.0	1.8	0.2	0.1	0.0	0.0	0.1	0.1	0.1	0.3	0.2	0.0	0.0	**1.2 ± 0.0**	**1.6 ± 0.1**	
			**1103**	0.1	0.0	1.1	0.3	1.1	0.3	0.7	0.1	0.2	0.2	0.3	0.6	0.5	0.2	0.1	0.4	**0.9 ± 0.1**	0.8 ± 0.1	0.6 ± 0.1
		Flavobacteriaceae	**1122**	0.1	0.2	1.4	0.3	2.7	1.3	0.7	0.0	0.1	0.2	0.7	0.7	1.7	1.1	0.2	0.0	**1.1 ± 0.3**	**1.7 ± 0.8**	0.6 ± 0.1
		Sphingobacteriaceae	**1107**	0.1	0.1	2.8	0.0	2.2	0.9	0.3	0.0	0.0	0.2	0.6	0.5	1.1	1.7	0.1	0.1	**2.5 ± 0.3**	**1.5 ± 0.5**	
		Chitinophagaceae	**1091**	0.2	0.9	4.0	0.9	2.2	1.5	1.4	0.4	0.1	0.9	0.9	2.0	0.0	3.1	0.5	0.5	**3.3 ± 0.4**	**1.3 ± 0.7**	**1.1 ± 0.2**
		**unclassified**	**1111**	0.1	0.1	0.1	0.5	1.0	0.4	0.3	0.5	0.0	0.1	1.0	0.9	0.3	0.6	0.0	0.5		**0.7 ± 0.1**	
Gemmatimonadetes		***Gemmatimonadaceae***	**400**	0.4	0.4	1.2	0.1	0.0	0.0	0.7	0.0	0.6	0.2	0.5	0.3	0.2	0.1	0.1	0.0	**0.9 ± 0.2**		
Planctomycetes		***Gemmataceae***	**934**	1.4	0.9	0.5	1.3	0.1	1.4	3.7	1.9	1.9	0.8	1.0	0.5	1.5	1.0	2.8	1.4			**1.7 ± 0.7**
			**944**	0.4	0.5	0.3	0.3	0.4	0.1	1.4	0.2	0.2	0.5	0.2	0.4	0.9	0.1	0.7	1.1			**1.0 ± 0.3**
		Tepidisphaeraceae	**985**	0.1	0.1	0.1	0.1	0.3	0.1	1.1	0.1	0.0	0.3	0.3	0.2	0.2	0.3	0.5	0.1			0.9 ± 0.2
		Phycisphaerae	**1033**	1.4	2.1	1.4	1.7	3.4	2.7	3.0	0.6	1.4	1.6	1.1	2.1	1.2	3.0	1.5	1.9		**1.7 ± 0.8**	**1.8 ± 0.5**
Proteobacteria	***α***	***Caulobacteraceae***	**653**	0.7	0.7	3.2	0.0	2.3	0.6	3.3	0.5	0.9	0.5	0.4	0.9	1.2	1.3	0.7	0.9	**2.9 ± 0.3**	**1.5 ± 0.3**	**2.6 ± 0.1**
	**β**	***Oxalobacteriaceae***	**332**	2.3	2.7	21.9	2.5	11.0	4.9	2.9	7.3	1.7	1.6	6.5	7.4	6.0	6.6	5.6	6.7	**18.2 ± 2.4**	**6.6 ± 1.9**	
	**γ**	***Pseudomonadaceae***	**310**	0.0	0.0	45.7	0.1	29.9	4.7	1.1	0.0	0.0	0.0	9.9	8.7	6.8	6.6	0.3	0.0	**42.3 ± 5.6**	**26.1 ± 3.5**	**1.0 ± 0.1**
	**δ**	***Bacteriovoracaceae***	**380**	0.0	0.0	0.1	0.0	1.9	0.1	3.9	0.1	0.0	0.0	0.2	0.0	0.8	0.3	0.1	0.2		**1.6 ± 0.5**	**3.8 ± 0.0**
		Bdellovibrionaceae	**779**	0.0	0.0	0.0	0.0	1.6	1.0	0.5	0.0	0.0	0.0	0.0	0.0	0.6	0.2	0.0	0.0		**1.1 ± 0.5**	
			**783**	0.1	0.1	0.0	0.1	1.6	0.5	5.0	0.4	0.0	0.0	0.0	0.1	0.4	0.9	0.1	0.4		**1.3 ± 0.2**	**4.8 ± 0.1**
		**unclassified**	**79**	0.0	0.0	0.0	0.0	5.1	0.6	13.7	0.3	0.0	0.0	0.0	0.0	1.7	1.0	0.6	1.2		**4.3 ± 0.9**	**13.4 ± 0.3**
Verrucomicrobia		**unclassified**	**868**	0.7	0.8	0.8	2.0	1.5	1.0	2.5	0.6	0.4	0.7	1.0	0.8	0.9	0.7	0.2	0.4			**2.0 ± 0.3**
		**unclassified**	**878**	0.0	0.1	0.0	0.0	0.3	0.1	4.8	0.1	0.0	0.0	0.0	0.0	0.2	0.1	0.2	0.3			**4.6 ± 0.1**
		Opitutaceae	**914**	0.0	0.1	0.2	0.0	1.1	0.6	0.6	0.1	0.0	0.1	0.0	0.3	0.2	0.8	0.0	0.0		0.8 ± 0.3	
		Verrucomicrobiaceae	**875**	0.0	0.0	0.0	0.0	1.2	0.2	0.2	0.0	0.0	0.0	0.0	0.0	0.6	0.6	0.1	0.1		1.0 ± 0.3	

[a] R_{CS} sores were calculated using the values for the relative abundance of a given OTU in the heavy (H) and light (L) fractions of [^{13}C]-chitin and [^{12}C]-chitin incubations. See materials and methods for details on the calculation.

Table S7. Relative abundances and calculated R_{CS} scores of labeled OTUs under anoxic conditions.[a]

Phylum	Family	OTU	[^{13}C]-Chitin Day 0 H	L	Day 10 H	L	Day 21 H	L	Day 70 H	L	[^{12}C]-Chitin Day 0 H	L	Day 10 H	L	Day 21 H	L	Day 70 H	L	R_{CS} score Day 10	Day 21	Day 70
Acidobacteria	*Acidobacteriaceae*	410	0.4	0.8	6.9	0.5	4.7	0.8	5.4	0.6	0.6	0.8	1.7	1.8	2.8	2.3	0.9	1.2	6.0 ± 0.7	3.4 ± 1.3	4.8 ± 0.3
Bacteroidetes	unclassified	754	0.0	0.0	0.9	0.0	27.3	0.1	35.7	2.7	0.0	0.0	0.0	0.1	8.0	13.7	7.6	16.4		24.6 ± 4.6	32.2 ± 3.8
Chloroflexi	*Anaerolineaceae*	547	0.1	0.8	0.6	0.5	1.4	0.4	2.3	0.6	0.0	0.1	0.1	0.1	0.6	0.4	0.1	0.4			2.0 ± 0.3
	Anaerolineaceae	548	0.6	0.3	2.5	1.2	1.0	0.1	0.0	0.0	0.6	0.3	0.4	0.8	0.4	0.3	0.0	0.1	1.8 ± 0.4		
	unclassified	530	2.6	2.4	3.8	3.3	1.9	2.9	4.2	1.9	3.1	2.9	3.1	3.4	3.7	3.3	3.1	3.1			1.6 ± 0.6
Firmicutes	*Paenibacillaceae*	203	0.9	1.0	5.2	1.9	12.9	1.7	15.1	1.7	2.2	1.3	1.6	1.4	1.3	1.7	2.0	1.8	3.7 ± 0.5	11.6 ± 0.4	13.5 ± 0.6
	unclassified	440	0.0	0.0	0.0	0.0	0.2	0.0	2.7	0.0	0.0	0.0	0.0	0.0	0.0	0.0	0.6	0.3			2.4 ± 0.3
	Lachnospiraceae	423	0.0	0.2	0.0	0.0	11.8	0.4	6.3	0.1	0.0	0.1	0.0	0.1	0.1	0.4	0.5	0.6		11.6 ± 0.2	6.1 ± 0.3
	Ruminococcaceae	416	0.0	0.2	0.3	0.0	15.6	0.7	2.2	0.3	0.1	0.0	0.4	0.1	0.4	0.4	1.6	1.0		15.2 ± 0.3	1.5 ± 0.8
Proteobacteria	*Geobacteraceae*	120	0.2	0.3	3.7	1.0	1.3	1.0	0.2	0.7	0.4	0.3	0.9	0.8	1.4	1.0	0.5	0.9	3.0 ± 0.4		
Verrucomicrobia	unclassified	2	1.0	1.2	2.1	0.6	1.1	0.9	0.6	0.5	1.0	0.6	0.5	1.2	1.1	1.3	0.5	0.6	1.4 ± 0.3		

[a]R_{CS} sores were calculated using the values for the relative abundance of a given OTU in the heavy (H) and light (L) fractions of [^{13}C]-chitin and [^{12}C]-chitin incubations. See materials and methods for details on the calculation.

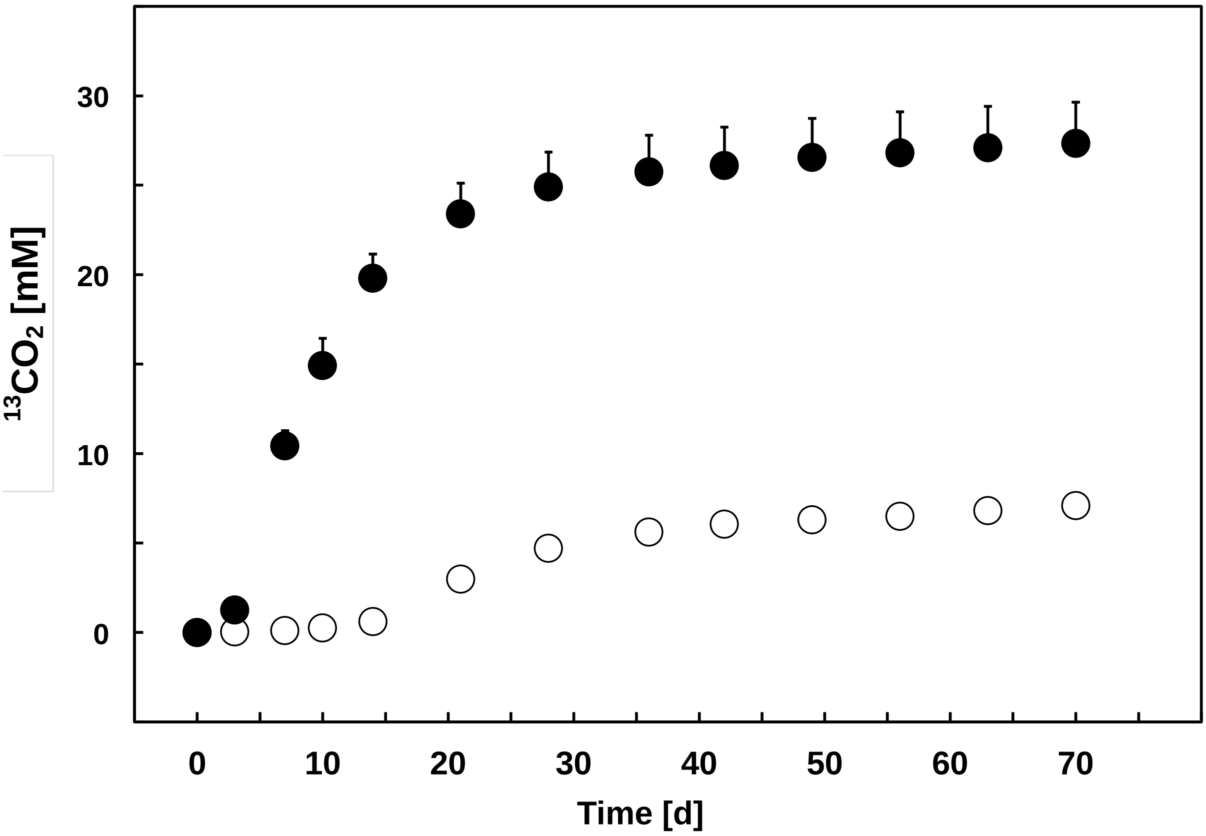

Figure S5. Evolution of $^{13}CO_2$ from [^{13}C]-chitin treatments under oxic (black circles) and anoxic (white circles) conditions. Error bars, standard deviation.

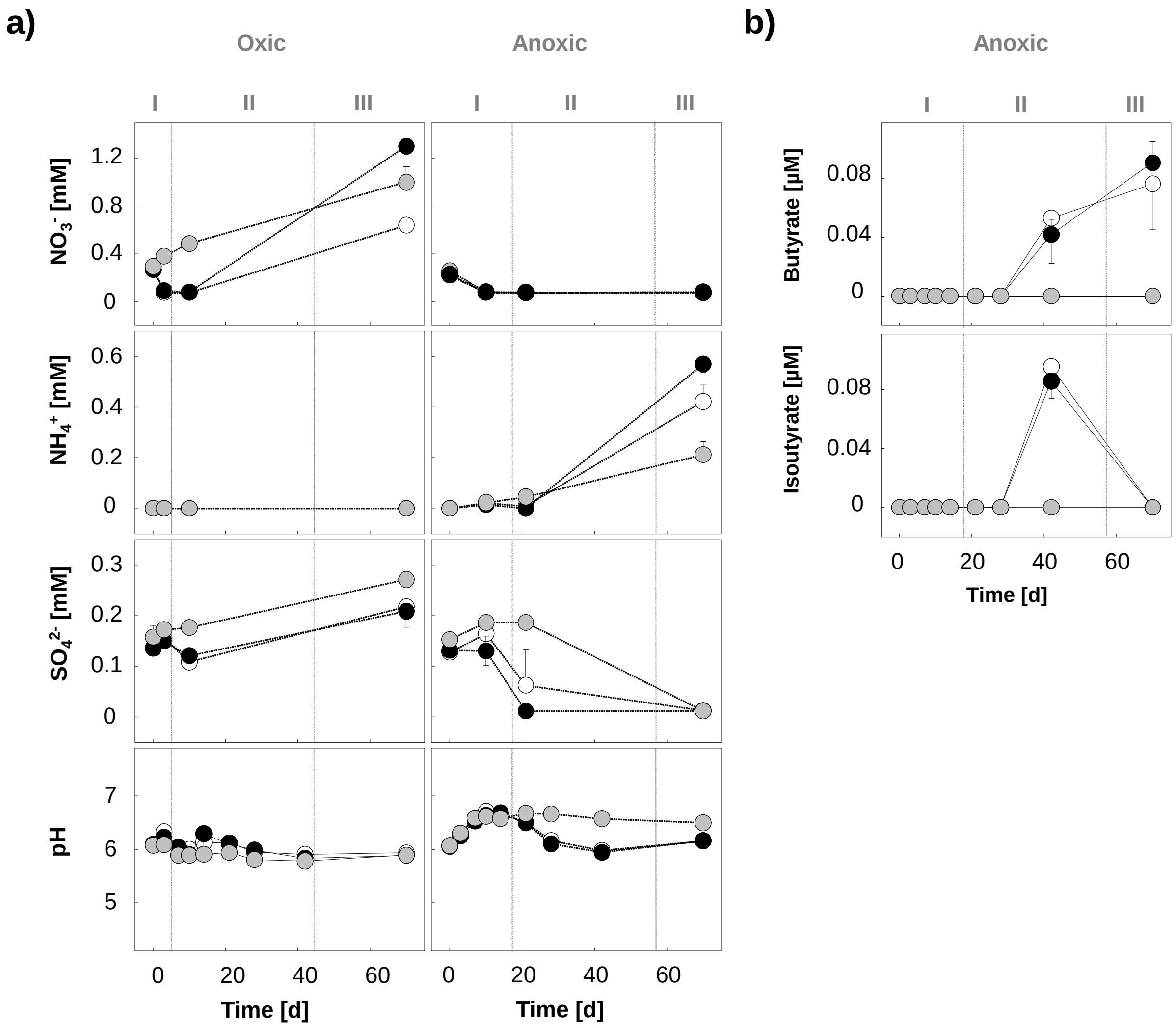

Figure S6. Product patterns of soil slurries supplemented with [^{13}C]-chitin, [^{12}C]-chitin and unsupplemented controls under oxic and anoxic conditions. (a) NO_3^-, NH_4^+ and SO_4^{2-} and pH. (b) organic compounds measured by HPLC (Figure 1b, major organic products and intermediates). Black circles, [^{13}C]-chitin treatments. White circles: [^{12}C]-chitin treatments. Grey circles, unsupplemented controls. Values are the means of duplicate microcosms. Error bars, standard deviation. Numerals, different phases during chitin degradation.

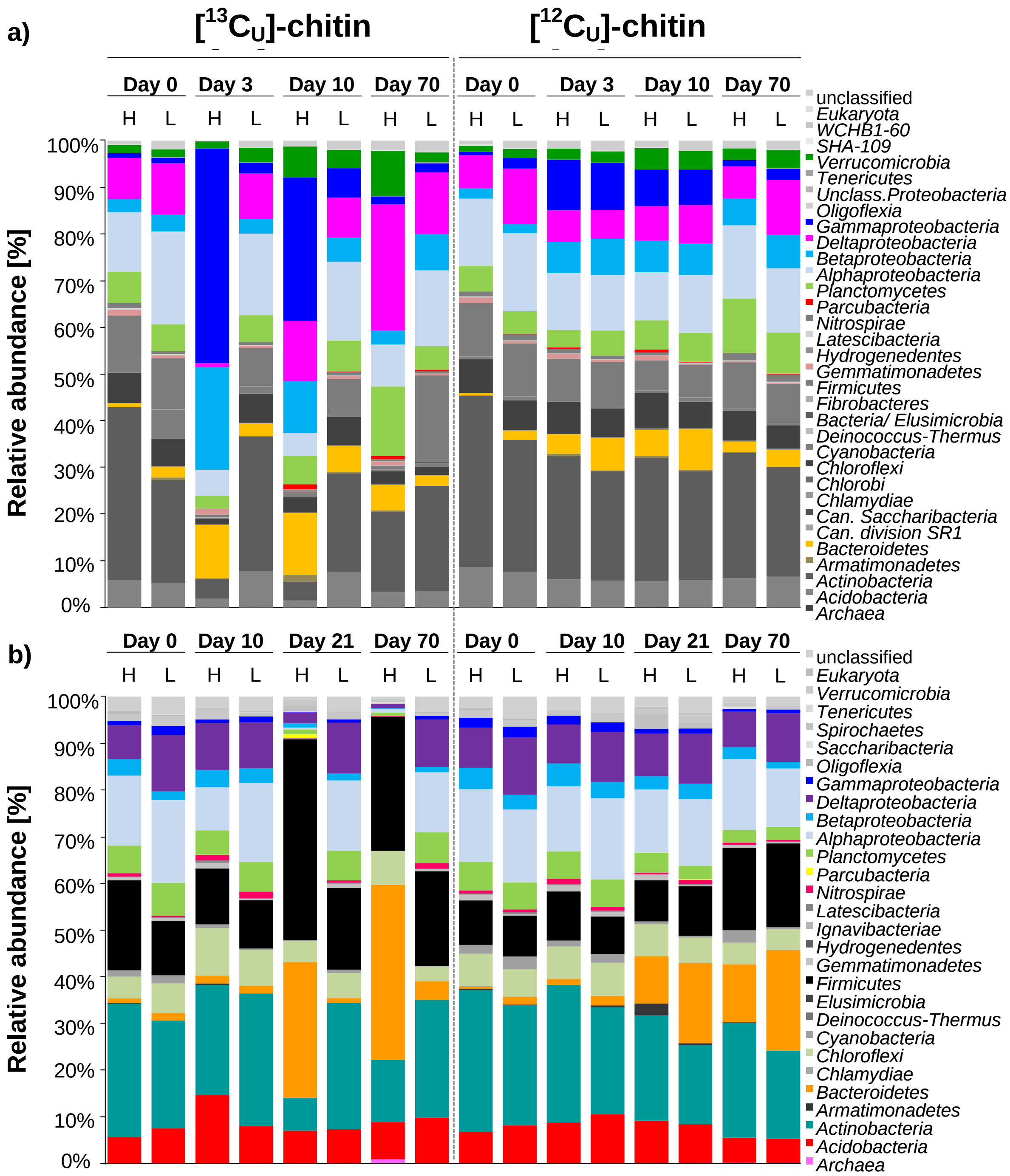

Figure S7. Microbial community composition based on 16S rRNA libraries derived from cDNA of heavy and light fractions after isopycnic centrifugation of RNA from [^{13}C]- and [^{12}C]-chitin soil slurry incubations under oxic (a) and anoxic (b) conditions. Jaguc2 (Nebel et al., 2011) was used to cluster pyrosequencing derived sequences into family level OTUs with sequence similarity threshold of 87.5% (Yarza et al., 2010). OTUs were phylogenetically affiliated by local nucleotide BLAST using Jaguc2 against the latest SILVA SSU database release.

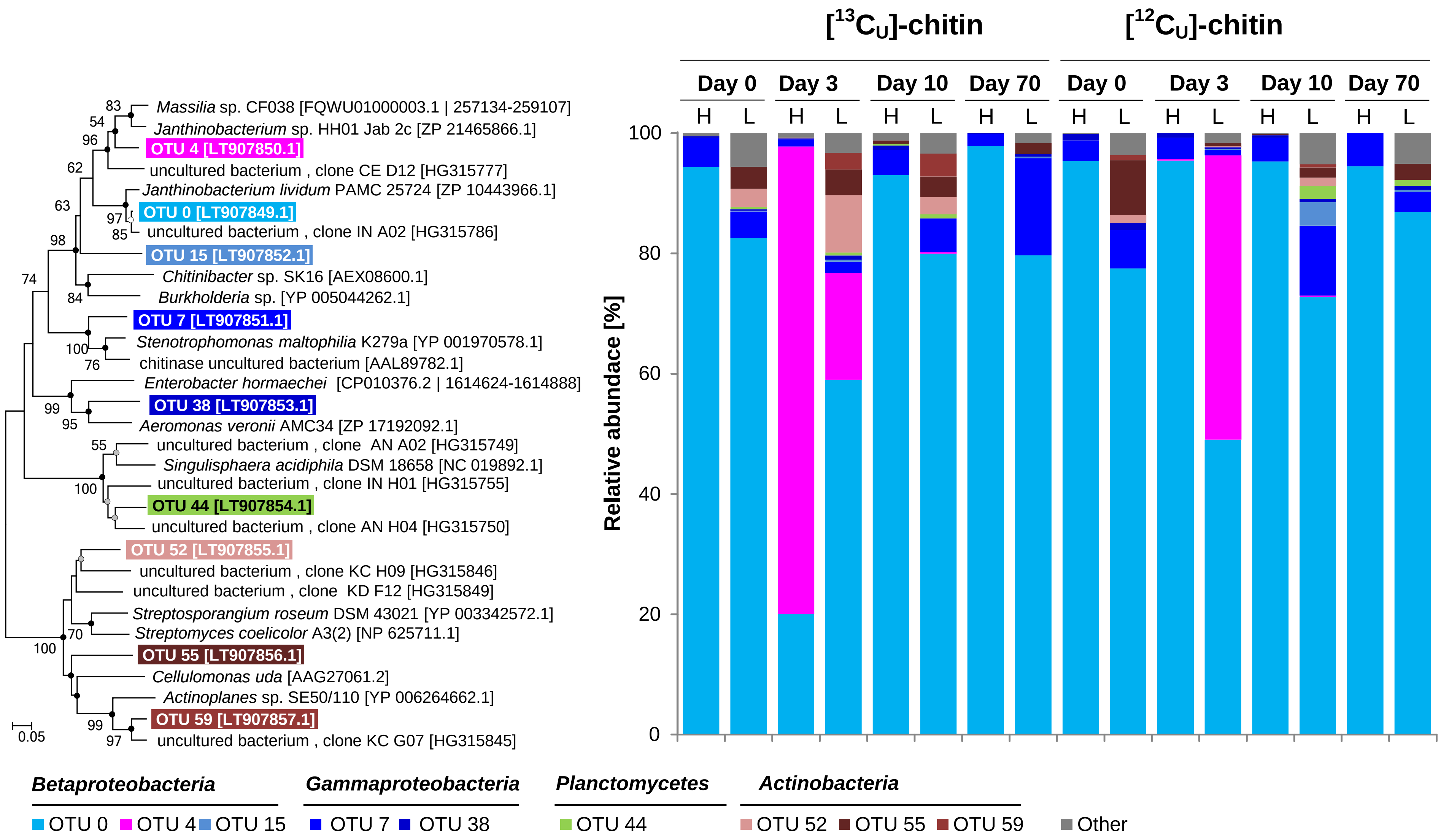

Figure S8. Phylogenetic tree and relative abundances of ChiA OTUs in the heavy and light RNA of [^{13}C]- and [^{12}C]-chitin treatments under oxic conditions. *ChiA* transcript libraries were prepared by Illumina-Amplicon sequencing of PCR-products from cDNA. Accession numbers of reference sequences are given in brackets. The tree was calculated using translated amino acid sequences with neighbor-joining algorithm (MEGA 6; Tamura et al., 2013) including bootstrapping (1,000 replicates; percentage values at nodes). Open circles and gray filled circles at nodes, these nodes were confirmed by maximum likelihood and maximum parsimony algorithms, respectively, using the same data set. Black circles, confirmation by both algorithms. Scale bar, 5% sequence divergence.

Reference

Tamura, K., Stecher, G., Peterson, D., Filipski, A., and Kumar, S. (2013). MEGA6: Molecular Evolutionary Genetics Analysis. Version 6.0. *Mol. Biol. Evol.* 30, 2725–2729. doi: 10.1093/molbev/mst197

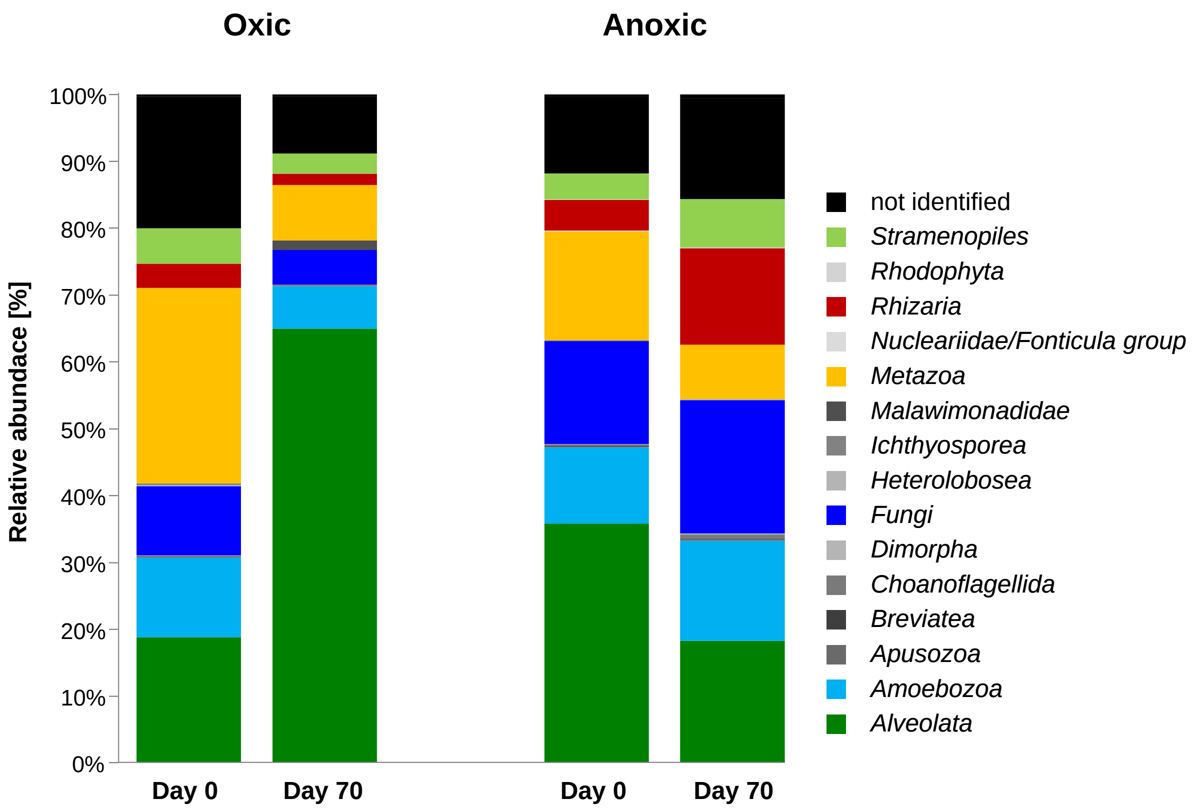

Figure S9. Detectable *Eukarya* at day 0 and after 70 days of incubation under oxic and anoxic conditions. Libraries were prepared from pooled PCR products derived from light fractions of [^{13}C]- and [^{12}C]-chitin treatments.

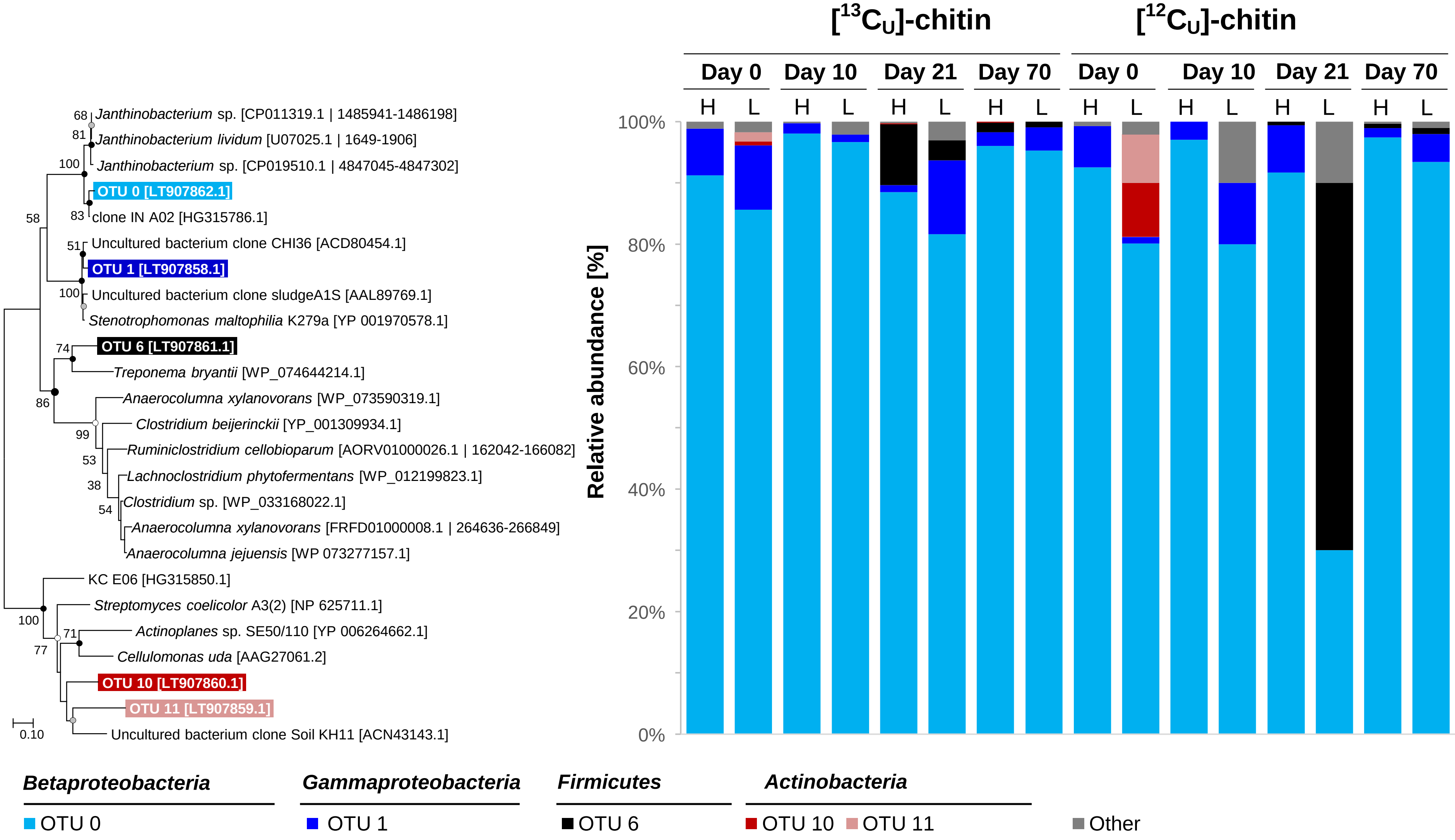

Figure S10. Phylogenetic tree and relative abundances of ChiA OTUs in the heavy and light RNA of [^{13}C]- and [^{12}C]-chitin treatments under anoxic conditions. *ChiA* transcript libraries were prepared by amplicon reads of PCR-products from cDNA. In brackets, Accession numbers of reference sequences. The tree was calculated using translated amino acid sequences with neighbor-joining algorithm (MEGA 6; Tamura et al., 2013) including bootstrapping (1,000 replicates; percentage values at nodes). Open circles and gray filled circles at nodes, these nodes were confirmed by maximum likelihood and maximum parsimony algorithms, respectively, using the same data set. Black circles, confirmation by both algorithms. Scale bar, 10% sequence divergence.

Reference

Tamura, K., Stecher, G., Peterson, D., Filipski, A., and Kumar, S. (2013). MEGA6: Molecular Evolutionary Genetics Analysis. Version 6.0. *Mol. Biol. Evol.* 30, 2725–2729. doi: 10.1093/molbev/mst197

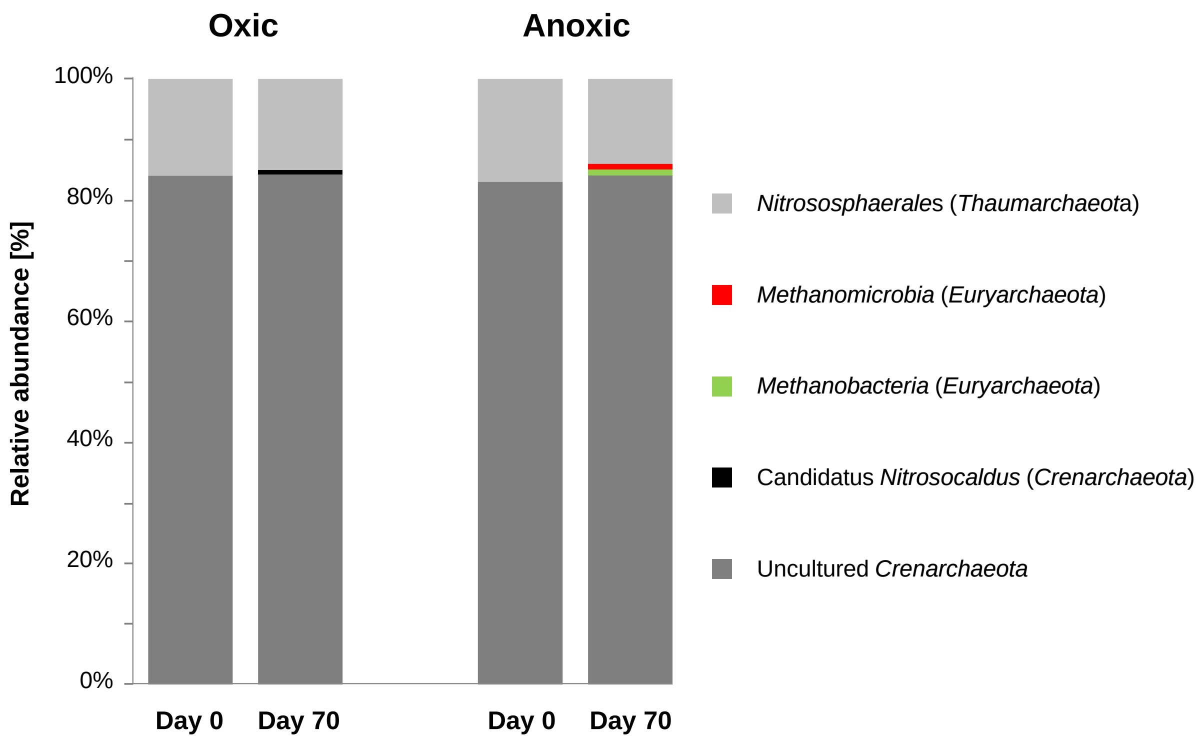

Figure S11. Relative abundance of detectable *Archaea* at day 0 and after 70 days of incubation under oxic and anoxic conditions. Libraries were prepared from pooled PCR products derived from light fractions of [^{13}C]- and [^{12}C]-chitin treatments.
